# SCP2 mediates the transport of lipid hydroperoxides to mitochondria in chondrocyte ferroptosis

**DOI:** 10.1038/s41420-023-01522-x

**Published:** 2023-07-08

**Authors:** Tianming Dai, Xiang Xue, Jian Huang, Zhenyu Yang, Pengfei Xu, Min Wang, Wuyan Xu, Zhencheng Feng, Weicong Zhu, Yangyang Xu, Junyan Chen, Siming Li, Qingqi Meng

**Affiliations:** 1 Guangzhou Institute of Traumatic Surgery, Guangzhou Red Cross Hospital of Jinan University, Guangzhou, 510220 China; 2 Department of Orthopedics, Guangzhou Red Cross Hospital of Jinan University, Guangzhou, 510220 China; 3 Department of Thoracic and Vascular Surgery, University Hospital Ulm, Albert-Einstein-Allee 23, 89081 Ulm, Germany; 4 Guizhou Medical University, Guiyang, 550025 China

**Keywords:** Cell death, Mitochondria, Lysosomes, Osteoarthritis, Chronic inflammation

## Abstract

Sterol carrier protein 2 (SCP2) is highly expressed in human osteoarthritis (OA) cartilage, accompanied by ferroptosis hallmarks, especially the accumulation of lipid hydroperoxides (LPO). However, the role of SCP2 in chondrocyte ferroptosis remains unexplored. Here, we identify that SCP2 transports cytoplasmic LPO to mitochondria in RSL3-induced chondrocyte ferroptosis, resulting in mitochondrial membrane damage and release of reactive oxygen species (ROS). The localization of SCP2 on mitochondria is associated with mitochondrial membrane potential, but independent of microtubules transport or voltage-dependent anion channel. Moreover, SCP2 promotes lysosomal LPO increase and lysosomal membrane damage through elevating ROS. However, SCP2 is not directly involved in the cell membrane rupture caused by RSL3. Inhibition of SCP2 markedly protects mitochondria and reduces LPO levels, attenuating chondrocyte ferroptosis in vitro and alleviating the progression of OA in rats. Our study demonstrates that SCP2 mediates the transport of cytoplasmic LPO to mitochondria and the spread of intracellular LPO, accelerating chondrocyte ferroptosis.

## Introduction

Osteoarthritis (OA) is a common chronic degenerative joint disease [1, 2], the pathogenesis of which remains obscure [3, 4]. The progression of OA is closely linked to cell death and the degradation of the extracellular matrix (ECM) [5–8]. The damaged chondrocytes in OA patients have been shown to display various types of cell death phenotypes such as necrosis, apoptosis, or pyroptosis, which makes these chondrocytes lose the ability to regulate ECM metabolism and maintain homeostasis in cartilage [9–11]. Therefore, regulating the viability of chondrocytes may play a key role in the development of OA.

Significant changes in the transcription of ferroptosis-related genes in human OA cartilage were found through data mining [12]. Combined with reports of iron accumulation and oxidative damage in OA, it has been suggested that ferroptosis may exist in OA [13–15]. Ferroptosis is a programmed cell death driven by iron-dependent lipid reactive oxygen species (ROS) [16]. When an intracellular iron overload occurs or the antioxidant system is abnormal, lipid hydroperoxides (LPO)—a form of lipid ROS produced by lipid metabolism—increase and damage the membrane of organelles such as mitochondria and cell membranes [17, 18]. Lysosomes plays an important role in the degradation of impaired mitochondria, but it is still unclear whether lysosomes can maintain normal function during ferroptosis [19]. In our study, ferroptosis was observed in human OA cartilage, with an increase in both iron and malondialdehyde (MDA) and a decrease in GPX4, especially the accumulation of LPO in OA primary chondrocytes. Nevertheless, how LPO are transported to organelles and promote ferroptosis remains largely unexplored [19, 20].

Interestingly, it has been found that the differentially expressed genes in human OA cartilage and our previous identification of differential proteins in synovial fluid with cartilage injury share a common up-regulated point—SCP2 (unreported in cartilage). Based on the above transcriptomic and proteomic clues, the high expression of SCP2 in human OA cartilage was further confirmed, suggesting that SCP2 may participate in cartilage damage. SCP2 is a nonspecific lipid transporter that can bind fatty acids, phospholipids, and other lipids [21, 22]. Hepatoma cells overexpressing SCP2 were found to be more susceptible to damage by the cholesterol hydroperoxide 7α-OOH (an exogenous LPO) relative to control cells [23]. Inhibition of SCP2 was also found to increase cell viability in GPX4^-/-^ fibroblasts or RSL3-treated breast cancer cells [24, 25]. Thus, we hypothesized that when chondrocytes undergo ferroptosis, the high expression of SCP2 may be involved in the transport of cytoplasmic LPO to organelles, which expedites membrane lipid peroxidation and organelle damage.

In the present study, whether the transport of cytoplasmic LPO to mitochondria and lysosomes via SCP2 was investigated in RSL3-treated rat chondrocytes, while the efficacy of the SCP2 inhibitor ScpI2 for relieving ferroptosis in OA cartilage was assessed in a rat OA model. This study also aimed to elucidate the relationship between SCP2-LPO-organelles during chondrocyte ferroptosis, as well as to provide an alternative therapeutic strategy for OA treatment in patients.

## Results

### Ferroptosis occurs in human OA cartilage with SCP2 increase

**Fig. 1:**
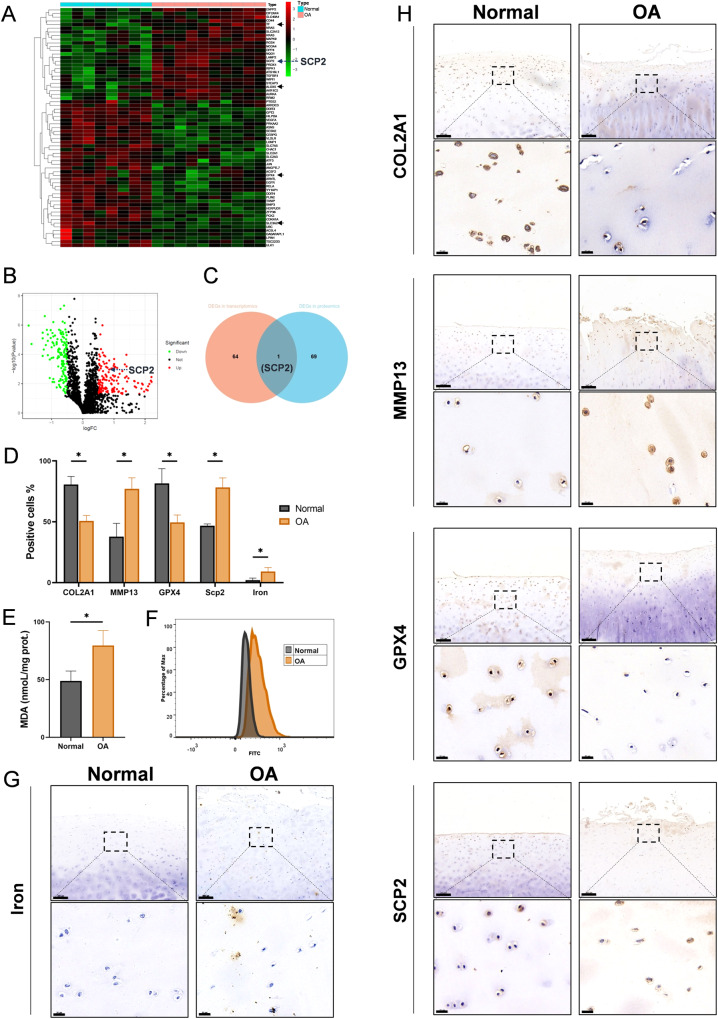
SCP2 expression is upregulated in human OA cartilage with ferroptosis. **A** Transcription levels of 66 ferroptosis-related differentially expressed genes in human OA cartilage (*n* = 10 per group) compared with those in normal cartilage (*n* = 8 per group). **B** The differentially expressed proteins in the synovial fluid from rabbit joints were analyzed by volcano plots between injured cartilage (*n* = 3 per group) and normal cartilage (*n* = 3 per group). **C** Venn diagram showed the overlapped DEGs between transcriptomics and proteomics. **D** Positive cell proportions (%) of immunohistochemistry staining and iron staining in human OA (*n* = 5 per group) and normal (*n* = 3 per group) cartilage. **E** MDA contents of human primary chondrocytes from OA (*n* = 5 per group) and normal (*n* = 3 per group) cartilage. **F** Flow cytometry analysis of lipid peroxides level of human primary chondrocytes from OA (*n* = 5 per group) and normal (*n* = 3 per group) cartilage. **G** Cartilage sections were stained with DAB-enhanced Prussian blue to detect iron in human OA (*n* = 5 per group) and normal (*n* = 3 per group) cartilage. Scale bar, 200 μm (top), 20 μm (bottom). **H** Immunohistochemistry staining of COL2A1, MMP13, GPX4, and SCP2 in human OA (*n* = 5 per group) and normal (*n* = 3 per group) cartilage. Scale bar, 200 μm (top), 20 μm (bottom). Data are expressed as means + SD. Unpaired two-tailed *t* tests, *P < 0.05.

By means of bioinformatics, transcriptomic analysis of ferroptosis-related differentially expressed genes (DEGs) showed that ferroptosis drivers such as *SCP2*, *ALOX5*, and *TF* were significantly up-regulated while suppressors such as *GPX4* and *SLC3A2* were significantly down-regulated in OA cartilage (Fig. 1A, Table S1). Combined with previous research in proteomics of the synovial fluid from rabbit joints, we found that the expression of SCP2 was significantly higher in synovial fluid with cartilage injury than that without injury (Fig. 1B, Fig. S1A–D), and SCP2 was the overlapped DEGs between transcriptomics and proteomics (Fig. 1C). To further explore whether ferroptosis was involved in OA progression with SCP2 increase, OA-related and ferroptosis-related indicators were assessed by immunohistochemistry in human cartilage as follows.

Cartilage tissues from patients who underwent total knee replacement (TKR) were designed as OA and cartilage from knee amputation surgery were designed as Normal (Table S2). The characteristics of OA were then confirmed by immunohistochemistry, which showed that the percentage of cells positive for COL2A1 was markedly decreased and the expression of MMP13 was significantly up-regulated in OA cartilage (Fig. 1D, H). Subsequently, indicators of ferroptosis were observed in both cartilage tissues and primary chondrocytes from these OA cartilage specimens. In OA cartilage, the positive cells for SCP2 and iron level were markedly elevated while the expression of GPX4 (a major anti-peroxidant enzyme) was markedly decreased (Fig. 1D, G, H). The MDA content and lipid peroxidation levels were also increased in OA primary chondrocytes (Fig. 1E, F), which indicated that lipid hydroperoxides (LPO) were accumulated in chondrocytes. In vitro, the cell viability of human primary chondrocytes from early-stage OA cartilage could be significantly improved by both the ferroptosis inhibitor Fer-1 and the SCP2 inhibitor ScpI2 (Fig. S2A). These findings suggested that ferroptosis occurred in human OA cartilage and SCP2 was involved in it.

### SCP2 regulates lipid peroxidation in RSL3-induced chondrocyte ferroptosis

**Fig. 2:**
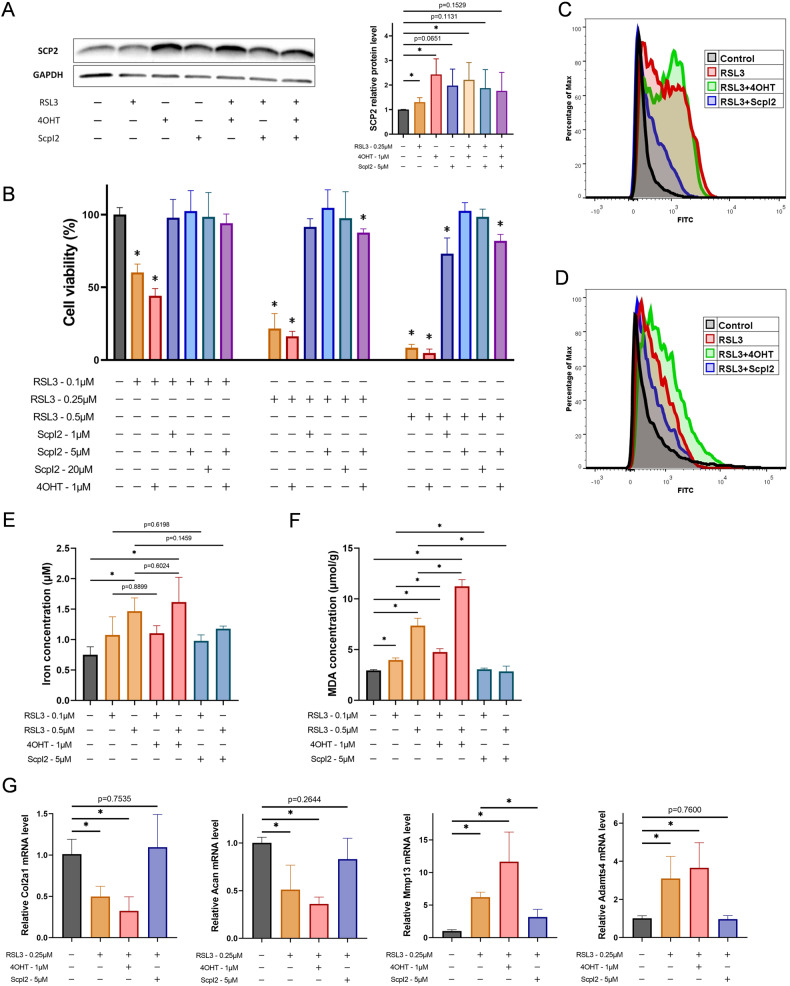
SCP2 regulates lipid peroxidation in RSL3-induced chondrocyte ferroptosis. **A** Western blot of SCP2 in rat chondrocytes after indicated treatment: 4OHT (1 μM) for 24 h to induce SCP2, then added RSL3 (0.25 μM) or ScpI2 (5 μM) for 4 h (*n* = 3 per group). **B** Chondrocyte viability was quantified by CCK-8 post indicated treatment: 4OHT (1 μM) was added 24 h before other treatment for induction of SCP2, then chondrocytes were treated with RSL3 and rescued by ScpI2 for 4 h (*n* = 3 per group). **C** Flow cytometry analysis of lipid peroxides level of rat chondrocytes after RSL3 (0.25 μM), 4OHT (1 μM), or ScpI2 (5 μM) treatment (*n* = 3 per group). **D** Flow cytometry analysis of ROS level of rat chondrocytes after RSL3 (0.25 μM), 4OHT (1 μM), or ScpI2 (5 μM) treatment (*n* = 3 per group). **E** Iron concentration of rat chondrocytes after RSL3, 4OHT, or ScpI2 treatment (*n* = 3 per group). **F** MDA concentration of rat chondrocytes after RSL3, 4OHT, or ScpI2 treatment (*n* = 3 per group). **G** Quantification of mRNA levels for Col2a1, Acan, Mmp13 and Adamts4 after RSL3, 4OHT, or ScpI2 treatment using qRT-PCR (*n* = 3 per group). Data are expressed as means + SD. For (**B**), compared to blank group. Unpaired two-tailed *t* tests, *P < 0.05.

Rat chondrocytes were utilized for studying the relationship between SCP2 and ferroptosis, while SCP2 inducer 4-hydroxy-tamoxifen (4OHT) or activity inhibitor ScpI2 was used for the regulation of SCP2. The expression of SCP2 increased in human OA cartilage, but whether SCP2 could be detected or induced by 4OHT in rat chondrocytes remains unknown. Thus, the expression of SCP2 in rat chondrocytes after RSL3, 4OHT or ScpI2 treatment was measured by Western blot. The results showed that the relative expression of SCP2 was significantly higher after RSL3, 4OHT, or RSL3 + 4OHT treatment than controls (Fig. 2A). SCP2 activity inhibitor ScpI2 did not reduce the protein expression of SCP2 (*P* > 0.05). Additionally, decreased expression of GPX4 after RSL3 or RSL3+ScpI2 treatment was observed (Fig. S2B).

The effects of RSL3, SCP2 inducer (4OHT), or inhibitor (ScpI2) on cell viability of rat chondrocyte were then investigated. When used alone, RSL3 could significantly reduce cell viability (Fig. 2B, orange column), whereas 4OHT or ScpI2 had no obvious effects on cell viability at the experimental concentration (Fig. S2C, D). A rescue study revealed that RSL3-induced cell viability decline (Fig. 2B, orange column) could be fully rescued by ScpI2 at a concentration of 5 μM (Fig. 2B, blue column), while the decrease of cell viability was aggravated after 4OHT treatment (Fig. 2B, red column).

We further assessed alterations in ferroptosis-related indicators. The results showed that intracellular iron levels were increased by RSL3 treatment, but they did not change significantly when co-treated with 4OHT or ScpI2 (Fig. 2E, *P* > 0.05). However, RSL3-induced accumulation of LPO (Fig. 2C) and ROS (Fig. 2D) was markedly restrained by ScpI2 or enhanced by 4OHT. Measurement of MDA content showed similar results as the LPO assay (Fig. 2F).

In RSL3-induced chondrocyte ferroptosis, the expression of cartilage anabolism related genes (Col2a1, Acan) decreased, while the catabolism related genes (MMP13, Adamts4) increased, and chondrocytes exhibited OA features (Fig. 2G). RSL3 + 4OHT or RSL3+ScpI2 exacerbated or attenuated these OA features, respectively, implicating that SCP2 also influenced the anabolism and catabolism of chondrocytes during ferroptosis (Fig. 2G).

These data suggested that SCP2 affected RSL3-induced chondrocyte ferroptosis and cartilage homeostasis mainly through regulating lipid peroxidation rather than iron, whereas the target organelles that SCP2 caused membrane lipid peroxidation are unclear.

### SCP2 regulates the membrane damage of organelles in chondrocyte ferroptosis

**Fig. 3:**
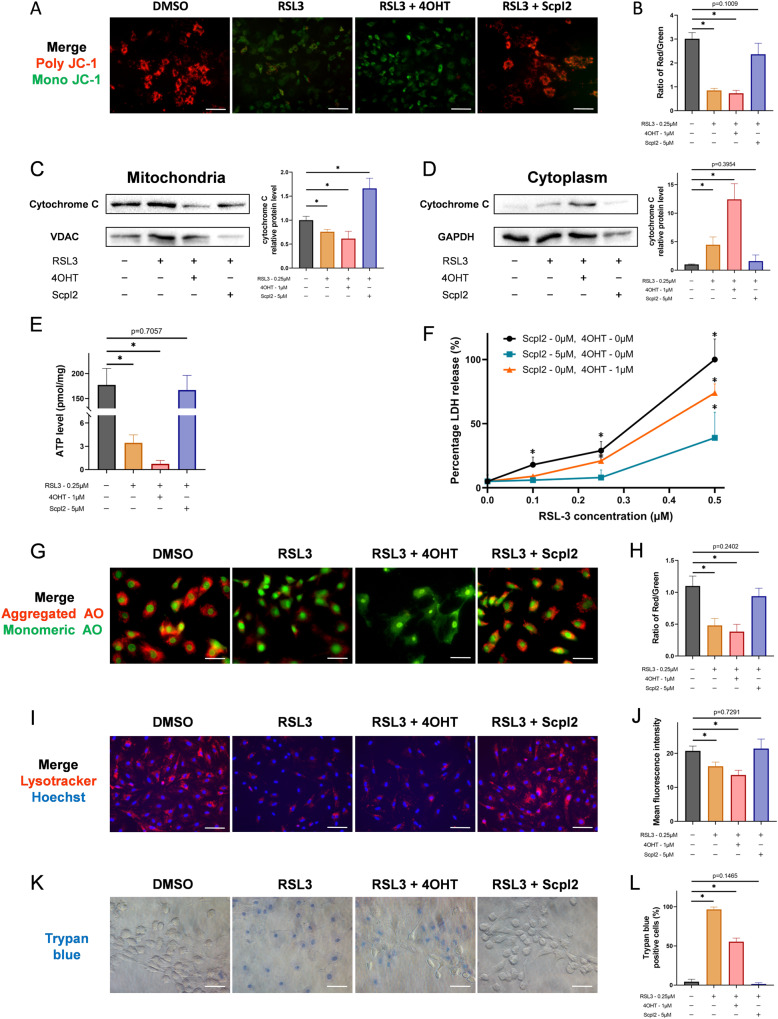
SCP2 mediates the membrane damage of mitochondria and lysosomes. **A** Mitochondrial membrane potential of rat chondrocytes treated with RSL3 (0.25 μM), 4OHT (1 μM), or ScpI2 (5 μM) detected by JC-1 staining. Scale bar, 100 μm. **B** The mean intensity ratio of red fluorescence to green fluorescence in JC-1 staining (*n* = 3 per group). **C** Western blot of cytochrome C and VDAC in mitochondria after RSL3, 4OHT, or ScpI2 treatment (*n* = 3 per group). **D** Western blot of cytochrome C and GAPDH in cytoplasm after RSL3, 4OHT, or ScpI2 treatment (*n* = 3 per group). **E** ATP levels of rat chondrocytes after RSL3, 4OHT, or ScpI2 treatment (*n* = 3 per group). **F** Percentage of LDH release (%) of rat chondrocytes after RSL3, 4OHT, or ScpI2 treatment (*n* = 3 per group). **G** Lysosomal membrane permeabilization of rat chondrocytes treated with RSL3 (0.25 μM), 4OHT (1 μM), or ScpI2 (5 μM) detected by acridine orange staining. Scale bar, 50 μm. **H** The mean intensity ratio of red fluorescence to green fluorescence in acridine orange staining (*n* = 3 per group). **I** Lysosomes staining in rat chondrocytes treated with RSL3 (0.25 μM), 4OHT (1 μM), or ScpI2 (5 μM) detected by lyso-tracker red. Scale bar, 100 μm. **J** The mean fluorescence intensity in lyso-tracker red staining (*n* = 3 per group). **K** Trypan blue staining of rat chondrocytes under RSL3 (0.25 μM), 4OHT (1 μM), or ScpI2 (5 μM) treatment. Scale bar, 50 μm. **L** Positive cell proportions (%) in trypan blue staining (*n* = 3 per group). Data are expressed as means + SD. Unpaired two-tailed *t* tests, *P < 0.05.

Since several organelles were reported to be damaged by LPO during ferroptosis [18], the main organelles where membrane damage mediated by SCP2 were then investigated in ferroptotic chondrocytes. For mitochondrial membrane damage, mitochondrial membrane potential (MMP), release of cytochrome C and ATP levels were measured. Poly JC-1 was dissociated into mono JC-1 when cells were treated with RSL3, indicating a decrease in MMP (Fig. 3A, B). The MMP almost returned to control levels after ScpI2 treatment, while 4OHT made the MMP even lower or completely abrogated it (Fig. 3A, B). The CCCP group served as the positive control, reflecting a strong decrease in MMP (Fig. S3C). Furthermore, the release of cytochrome C from impaired mitochondria (Fig. 3C) into cytoplasm (Fig. 3D) was found in the RSL3 and RSL3 + 4OHT groups, with the relative content of cytochrome C/VDAC on mitochondria decrease, and the relative content of cytochrome C/GAPDH in cytoplasm increase. This phenomenon was opposite in the RSL3+ScpI2 group. Similar trends were also observed at the ATP levels, which were low in the RSL3 and RSL3 + 4OHT groups, but restored in the RSL3+ScpI2 group (Fig. 3E).

Lysosomes plays an important role in the degradation of impaired mitochondria. Whether lysosomes would be damaged by SCP2 in RSL3-induced ferroptosis was then examined by acridine orange staining and lyso-tracker. We found that acridine orange emitted red fluorescence when aggregating in lysosomes in the control and RSL3+ScpI2 groups, suggesting that the lysosomal membrane was intact (Fig. 3G, H). In contrast, the lysosomal membrane permeabilization (LMP) increased in the RSL3 and RSL3 + 4OHT groups, leading to the reduction of red fluorescence and the enhancement of green fluorescence in the cytosol (Fig. 3G, H). Similarly, the mean fluorescence intensity of the lyso-tracker was much stronger in the control and RSL3+ScpI2 groups than in the RSL3 and RSL3 + 4OHT groups (Fig. 3I, J).

Cell membrane rupture occurs at the end of ferroptosis. Concerning the cell membrane, the results of the LDH release assay showed that RSL3-induced cell membrane rupture could be partially prevented by ScpI2 but not aggravated by 4OHT (Fig. 3F), suggesting there might be other factors driving cell membrane damage. Similarly, severe cell membrane rupture was found by the trypan blue staining in the RSL3 group with approximately 96% positive staining cells, while it was declined in the RSL3 + 4OHT and RSL3+ScpI2 groups (Fig. 3K, L).

The above data inferred that SCP2 was involved in mitochondrial and lysosomal membrane damage during ferroptosis, but SCP2 did not appear to be the main factor for damaging cell membranes.

### SCP2 mediates mitochondrial lipid peroxidation through transporting cytoplasmic LPO to mitochondria

**Fig. 4:**
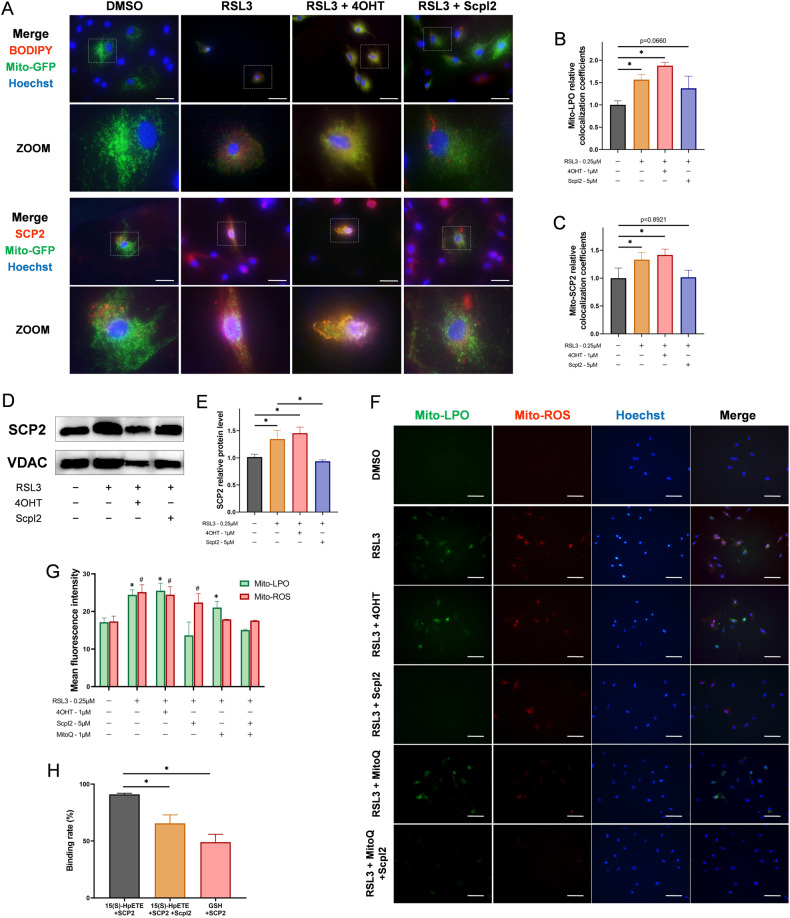
SCP2 transports cytoplasmic LPO to mitochondria. **A** The merged images of BODIPY 665/676, CellLight-mito-GFP, and Hoechst 33258 staining in rat chondrocytes treated with RSL3 (0.25 μM), 4OHT (1 μM), or ScpI2 (5 μM) were shown in the upper part. The merged images of immunofluorescence SCP2, CellLight-mito-GFP, and Hoechst 33258 staining in rat chondrocytes were shown in the lower part. Scale bar, 50 μm. **B** The relative colocalization coefficients of mitochondria and LPO with mito-GFP and BODIPY staining (*n* = 6 per group). **C** The relative colocalization coefficients of mitochondria and SCP2 with mito-GFP and immunofluorescence staining (*n* = 6 per group). **D** Western blot of SCP2 and VDAC in mitochondria after treatment of RSL3 (0.25 μM), 4OHT (1 μM), or ScpI2 (5 μM) (*n* = 3 per group). **E** SCP2 relative protein level in mitochondria after indicated treatment (*n* = 3 per group). **F** The mito-LPO was stained with Mito-PeDPP, and mito-ROS was stained with MitoSox in rat chondrocytes after RSL3 (0.25 μM), 4OHT (1 μM), MitoQ (1 μM), or ScpI2 (5 μM) treatment. Scale bar, 100 μm. **G** The mean fluorescence intensity for Mito-LPO staining (compared to blank group of Mito-LPO, *P < 0.05), and Mito-ROS staining (compared to blank group of Mito-ROS, ^#^*P* < 0.05), *n* = 6 per group. **H** The binding rates of SCP2 to 15(S)-HpETE or GSH were detected in phosphate buffered saline by LC-MS/MS (*n* = 3 per group). Unpaired two-tailed *t* tests, *P < 0.05.

Following the above, we investigated how SCP2 causes lipid peroxidation damage in mitochondria. Colocalization experiments of mitochondria-LPO and mitochondria-SCP2 demonstrated that LPO and SCP2 began to localize in mitochondria during RSL3-induced ferroptosis, while LPO and SCP2 on mitochondria continued to increase after RSL3 + 4OHT treatment, and their colocalization with mitochondria decreased synchronously in the RSL3+ScpI2 group (Fig. 4A–C). The results of the western blot revealed that the relative content of SCP2/VDAC on mitochondria increase after RSL3 or RSL3 + 4OHT treatment, and then decreased after adding ScpI2 (Fig. 4D, E). We speculated that SCP2 may bind and carry cytoplasmic LPO to mitochondria during ferroptosis, leading to the accumulation of mitochondrial LPO and membrane impairment.

The formation of mitochondrial LPO may be related to the transfer of cytoplasmic LPO or the generation from mitochondrial ROS. To further identify the source of mitochondrial LPO, the mitochondrial LPO was labeled with MitoPeDPP (a mitochondrial LPO specific fluorescent probe), and mitochondrial ROS was assessed by MitoSox. We validated that the level of mitochondrial LPO changed with SCP2 inducer (4OHT) or inhibitor (ScpI2) in RSL3-induced ferroptosis (Fig. 4F, G), which was consistent with the colocalization experiment of mitochondria-LPO in Fig. 4A. In addition, we found that the mitochondrial ROS levels increased in the RSL3 and RSL3 + 4OHT groups (Fig. 4F, G). In the RSL3+MitoQ group, mitochondrial LPO was still present when mitochondrial ROS has been removed by MitoQ, and mitochondrial LPO disappeared when further adding ScpI2 (Fig. 4F, G). The RSL3+ScpI2 group inferred that, if ScpI2 was applied, the level of mitochondrial LPO became very low, but mitochondrial ROS still existed at this time, demonstrating that the accumulation of mitochondrial LPO mainly depended on SCP2 rather than generation from mitochondrial ROS (Fig. 4F, G).

Meanwhile, the colocalization of cytoplasmic LPO and mitochondria in the RSL3+MitoQ and RSL3+MitoQ+ScpI2 groups were also investigated. The results showed that cytoplasmic LPO was present in these two groups, while the colocalization of LPO and mitochondria was high after MitoQ treatment, but the colocalization decreased significantly after adding ScpI2 with the separation of red and green fluorescence (Fig. S3A, first and second lines). The above results suggested that SCP2 was more possible to transport cytoplasmic LPO to mitochondria, rather than affecting the production of mitochondrial LPO from ROS.

To explore whether SCP2 transported LPO through microtubules or voltage-dependent anion channel (VDAC), colchicine was used to interfere with microtubule formation, and DIDS was used to inhibit VDAC in the outer mitochondrial membrane. However, the results indicated that both colchicine and DIDS had no significant effect on the colocalization of LPO or SCP2 on mitochondria after RSL3 treatment (Fig. S3A, B, D, E), suggesting that the transport of LPO to mitochondria by SCP2 was independent of microtubules and VDAC. Interestingly, after treatment with the MMP destroyer CCCP, there was no significant increase in intracellular LPO, but a significant increase in the colocalization of SCP2 on mitochondria was found, suggesting that the decrease in MMP appeared to be another factor that recruited SCP2 to mitochondria (Fig. S3B, E).

Subsequently, the binding rate of SCP2 to 15(S)-HpETE (a typical lipid hydroperoxide in cells) was determined in vitro. The results revealed that SCP2 could bind strongly to 15(S)-HpETE, but the binding rate was markedly decreased after adding ScpI2 (Fig. 4H). Compared with glutathione (GSH, serve as negative control), the binding rate of SCP2 to GSH was much lower (Fig. 4H).

On the basis of these data, SCP2 accelerated the spread of LPO from the cytoplasm to mitochondria, causing mitochondrial membrane damage and then promoting the release of mitochondrial ROS, which may further affect other organelles.

### Lysosomal lipid peroxidation was related to ROS but independent of direct transport of LPO by SCP2

**Fig. 5:**
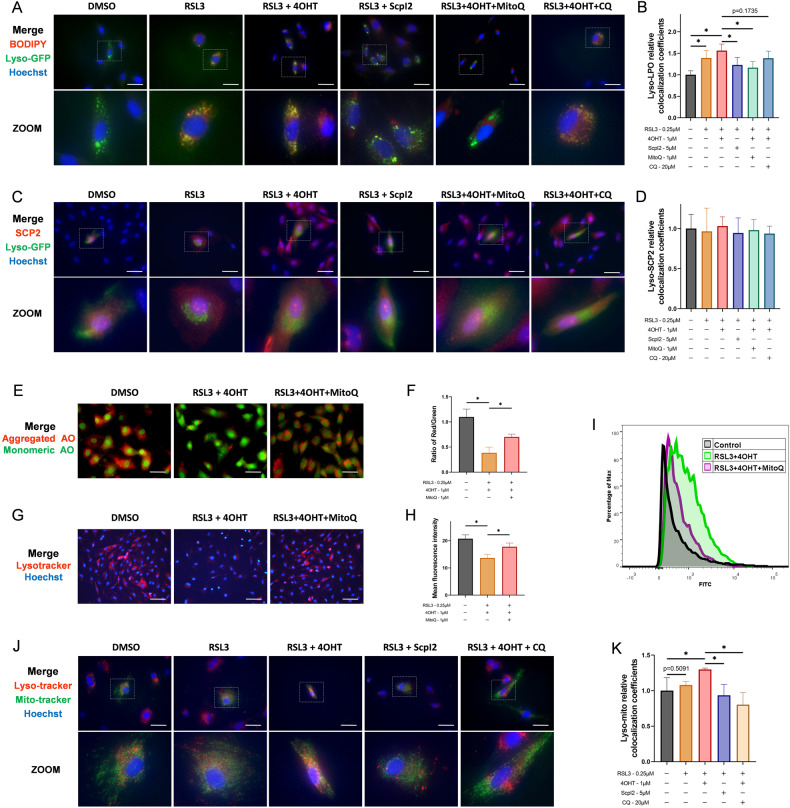
Lysosomal lipid peroxidation was related to ROS but independent of the direct transport of LPO by SCP2. **A** The merged images of BODIPY 665/676, CellLight-lyso-GFP, and Hoechst 33258 staining in rat chondrocytes treated with RSL3 (0.25 μM), 4OHT (1 μM), ScpI2 (5 μM), MitoQ (1 μM) or CQ (20 μM). Scale bar, 50 μm. **B** The relative colocalization coefficients of lysosomes and LPO with lyso-GFP and BODIPY staining (*n* = 6 per group). **C** The merged images of immunofluorescence SCP2, CellLight-lyso-GFP, and Hoechst 33258 staining in rat chondrocytes treated with RSL3 (0.25 μM), 4OHT (1 μM), ScpI2 (5 μM), MitoQ (1 μM) or CQ (20 μM). Scale bar, 50 μm. **D** The relative colocalization coefficients of lysosomes and SCP2 with lyso-GFP and immunofluorescence staining (*n* = 6 per group). **E** Lysosomal membrane permeabilization of rat chondrocytes treated with RSL3 (0.25 μM), 4OHT (1 μM), or MitoQ (1 μM) detected by acridine orange staining. Scale bar, 50 μm. **F** The mean intensity ratio of red fluorescence to green fluorescence in acridine orange staining (*n* = 3 per group). **G** Lysosomes staining in rat chondrocytes treated with RSL3 (0.25 μM), 4OHT (1 μM), or MitoQ (1 μM) detected by lyso-tracker red. Scale bar, 100 μm. **H** The mean fluorescence intensity in lyso-tracker red staining (*n* = 3 per group). **I** Flow cytometry analysis of ROS level of rat chondrocytes after RSL3 (0.25 μM), 4OHT (1 μM), or MitoQ (1 μM) treatment (*n* = 3 per group). **J** The merged images of Lyso-tracker red, Mito-tracker green, and Hoechst 33258 staining in rat chondrocytes treated with RSL3 (0.25 μM), 4OHT (1 μM), ScpI2 (5 μM), or CQ (20 μM). Scale bar, 50 μm. **K** The relative colocalization coefficients of mitochondria and lysosomes with Mito-tracker green and Lyso-tracker red staining (*n* = 6 per group). Data are expressed as means + SD. Unpaired two-tailed *t* tests, *P < 0.05.

How SCP2 damaged lysosomal membranes was then investigated. Colocalization experiments of lysosome-LPO demonstrated that LPO localized in lysosomes in RSL3-induced ferroptosis, while the LPO on lysosomes increased after RSL3 + 4OHT treatment, and the colocalization decreased in the RSL3+ScpI2 group (Fig. 5A, B). However, no significant change in the colocalization of lysosomes-SCP2 was observed between the control, RSL3, RSL3 + 4OHT, or RSL3+ScpI2 groups (Fig. 5C, D), which suggested that the increase of lysosomal LPO was not directly transported by SCP2. In addition, the LPO on lysosomes significantly decreased by adding MitoQ (Fig. 5A, B). Further measurement of LMP (AO staining) and lyso-tracker revealed that MitoQ could protect lysosomes from lipid peroxidation damage mediated by SCP2 (Fig. 5E–H). Meanwhile, the reduction of intracellular ROS (Fig. 5I) and the mitochondrial ROS (Fig. 4F, G) was observed after MitoQ treatment, indicating that the ROS released from impaired mitochondria induced by SCP2 may be the medium causing lysosomal lipid peroxidation.

Considering that damaged mitochondria with high levels of LPO and ROS may be engulfed by lysosomes and propagate lipid peroxidation, the colocalization of mitochondria-lysosomes was then investigated, and the autophagy inhibitor chloroquine (CQ) was employed [26]. Our results showed that the colocalization of mitochondria-lysosomes increased after RSL3 + 4OHT treatment, and significantly decreased in the RSL3+ScpI2 and RSL3 + 4OHT + CQ groups (Fig. 5J, K). This indicated that lysosome-mediated phagocytosis was enhanced with the increase of SCP2, and the phagocytosis could be reversed by CQ. Nevertheless, CQ intervention did not reduce the colocalization of lysosomes-LPO in RSL3-induced ferroptosis (Fig. 5A, B), nor the colocalization of lysosomes-SCP2 (Fig. 5C, D). As a result, lysosomal LPO was not caused by phagocytosis of damaged mitochondria.

### Suppression of SCP2 alleviates OA progression in a modified-Hulth rat model

**Fig. 6:**
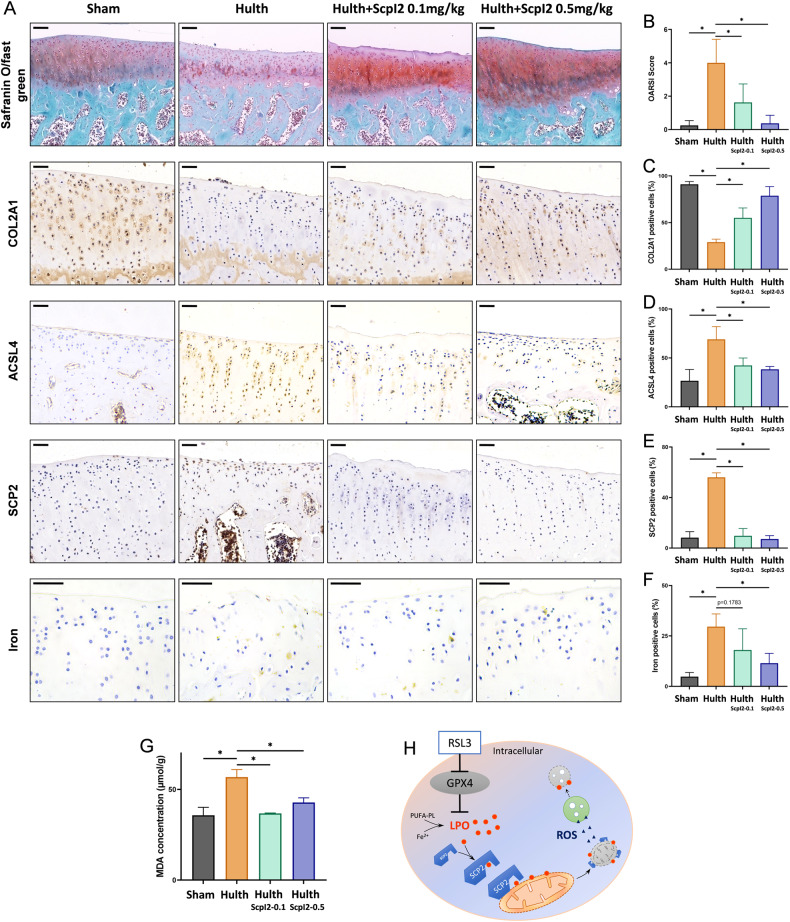
Suppression of SCP2 alleviates OA progression in a modified-Hulth rat model. **A** Experimental OA was examined by safranin O/fast green, prussian blue (with DAB), immunohistochemistry staining of COL2A1, ACSL4, and SCP2 in the articular cartilage of rats at 5 weeks after surgery (*n* = 4 per group). Scale bar, 100 μm for safranin O/fast green, 50 μm for the others. **B** OARSI score based on the results of safranin O/fast green staining (*n* = 4 per group). **C** Positive cell proportions (%) of immunohistochemistry staining of COL2A1 in rats' articular cartilage (*n* = 4 per group). **D** Positive cell proportions (%) of immunohistochemistry staining of ACSL4 in rats' articular cartilage (*n* = 4 per group). **E** Positive cell proportions (%) of immunohistochemistry staining of SCP2 in rats' articular cartilage (*n* = 4 per group). **F** Positive cell proportions (%) of iron staining in rats' articular cartilage (*n* = 4 per group). **G** MDA concentration of rat articular cartilage (*n* = 4 per group). **H** The schematic diagram for SCP2 mediating the transport of lipid peroxides to mitochondria and causing secondary damage to lysosomes during ferroptosis. Data are expressed as means + SD. Unpaired two-tailed *t* tests, *P < 0.05.

To explore the effect of the SCP2 activity inhibitor ScpI2 on OA progression, a modified-Hulth model was established in female SD rats. Cartilage degradation in the Hulth group was obvious, as evidenced by safranin O/fast green staining and higher OARSI scores (Fig. 6A, B, Fig. S4). In addition, cartilage degradation was also assessed by immunohistochemistry staining for Collagen type II (COL2A1). The number of COL2A1 positive cells in the Hulth group was reduced by threefold compared with that in the Sham group (Fig. 6A, C). The increase of positive cells for ACSL4, iron and SCP2 in the Hulth group suggested the occurrence of ferroptosis (Fig. 6A, D–F).

However, cartilage degradation was alleviated in the Hulth+ScpI2 groups through the suppression of SCP2, together with a significantly decreased OARSI score (Fig. 6A, B). Moreover, ScpI2 treatment significantly restored the expression of the matrix protein COL2A1 and decreased the level of iron as well as the ferroptosis-related proteins ACSL4 and SCP2 in the Hulth+ScpI2 group (Fig. 6A, C–F). An accumulation of MDA in the cartilage of the Hulth model was observed, while ScpI2 could reduce the MDA level in the Hulth+ScpI2 group (Fig. 6G). These findings implied that SCP2 activity inhibitor ScpI2 alleviated OA progression mainly by suppressing ferroptosis.

## Discussion

In recent years, changes in ferroptosis-related markers such as iron content and GPX4 expression have been reported in the cartilage from OA patients or mouse OA models [12–15]. Notably, we found that the levels of lipid peroxidation, the core of ferroptosis, were significantly higher in human primary OA chondrocytes than in normal chondrocytes, which further proved that ferroptosis occurs in human OA cartilage. In addition, by taking the intersection of the differentially expressed genes from the transcriptome and proteome, we found that SCP2 in injured cartilage was significantly up-regulated at both the mRNA and protein levels. Combined with the result of immunohistochemistry in the human OA cartilage and the rescue study of human OA chondrocytes, it seemed that SCP2 participated in the ferroptosis of OA cartilage. As we know, inhibition of SCP2 increases cell viability in GPX4^-/-^ fibroblasts or RSL3-treated breast cancer cells, but the mechanism driving this remains obscure [24, 25, 27]. Since SCP2 is a carrier protein that can carry a variety of lipids (including lipid hydroperoxides), the elevation of SCP2 was likely related to the transport of LPO in OA chondrocytes [21–23].

In ferroptosis, the imbalance of redox homeostasis, iron handling, or lipid metabolism is known to cause downstream LPO accumulation, and the initial lipid peroxidation step occurs in the cytoplasm outside the mitochondrial matrix [24]. However, it is unclear how cytoplasmic LPO damages organelles and leads to cell death [17, 19]. Based on our previous findings, we speculated that SCP2 may play a role in prompting LPO to disrupt the membrane system of organelles under RSL3 treatment. Apart from the hallmark events that destruction of the mitochondrial membrane and cell membrane in ferroptosis, damage to other organelle membrane systems has rarely been reported [16, 19, 27]. Therefore, the specific organelles with membrane damage mediated by SCP2 were sought in ferroptosis. It was found that SCP2 was involved not only in mitochondrial membrane damage but also in lysosomal membrane damage. In addition to iron or nitric oxide accumulation in lysosomes upon ferroptosis in literature [28, 29], membrane permeability alteration was observed in lysosomes which was regulated by SCP2. Surprisingly, cell membrane damage in ferroptosis did not appear to be dominated by SCP2, though SCP2 acts as a carrier for sterols to transport between membranes [30]. Collectively, SCP2 was shown to be involved in the damage of mitochondria and lysosomes in chondrocyte ferroptosis, which may cause cellular energy depletion and a release of ROS, expediting cell death [18].

To reveal the role of SCP2 on mitochondria and lysosomes, we investigated whether SCP2 delivers cytoplasmic LPO to mitochondria and lysosomes, thereby causing damage to both organelles. The results of western blot and colocalization demonstrated that LPO and SCP2 increased or decreased synchronously upon mitochondria. MitoPeDPP, a specific label for mitochondrial LPO, further confirmed that mitochondrial LPO was mainly regulated by SCP2. As has been discussed previously [24, 31], there are two main ways for the formation of mitochondrial LPO: the transfer of cytoplasmic LPO to mitochondria; or the generation of LPO from mitochondrial ROS. It has also been observed that mitochondrial LPO was still present after removing mitochondrial ROS using MitoQ, whereas mitochondrial LPO disappeared when SCP2 was inhibited, indicating that mitochondrial LPO was mainly transported from the cytoplasm by SCP2 after RSL3 treatment.

For lysosomes, LPO and SCP2 exhibited asynchronous changes in lysosomes during ferroptosis, which suggested that lysosomal LPO was not directly transported by SCP2. Further studies found that SCP2 enhanced the phagocytosis of lipid-peroxidized mitochondria by lysosomes. However, the autophagy inhibitor CQ failed to reduce lysosomal LPO, indicating that lysosomal LPO was not derived from the phagocytosis of lipid-peroxidized mitochondria [26]. It has also been reported that lysosomal LPO is affected by intracellular ROS levels and iron concentrations, which promotes lysosomal membrane permeabilization [32, 33]. Meanwhile, we found that MitoQ could decrease the LPO on lysosomes and maintain the integrity of lysosomal membranes, suggesting that mitochondrial ROS may be the medium causing lysosomal lipid peroxidation. Given the above results, SCP2 could significantly elevate mitochondrial ROS and intracellular ROS levels but not iron concentrations, inferring that the ROS released from impaired mitochondria after SCP2 damaged the mitochondrial membrane, causing secondary damage to lysosomes [34, 35]. Based on these data, SCP2 accelerated the spread of LPO and ROS to organelles, especially causing damage to mitochondria and lysosomes.

Regarding the transport of LPO to mitochondria by SCP2, microtubule-based transport is an important way of directional transport of intracellular substances [36]. By intervening in microtubule synthesis, no significant change was observed in the colocalization of SCP2-LPO-mitochondria after RSL3 treatment. Therefore, it can be inferred that microtubules did not play a major role in transporting LPO by SCP2. Moreover, it has been reported that VDAC is involved in ferroptosis which allows iron or fatty acid to enter mitochondria [27, 37], but the VDAC inhibitor DIDS could not prevent LPO and SCP2 from locating on mitochondria, suggesting that VDAC may not be a target of SCP2.

Interestingly, CCCP drove the localization of SCP2 to mitochondria when CCCP destroyed the MMP, which we ponder it may be a self-rescue process for cells to repair mitochondrial membrane through transferring non-oxidized phospholipids to mitochondria by SCP2 [21, 38]. Upon ferroptosis, the decrease of MMP induced LPO instead of normal phospholipids to be transported to mitochondria by SCP2, leading to the aggravation of mitochondrial membrane damage. This indicated that the decrease in MMP may be another factor that recruits SCP2 to mitochondria in addition to intracellular LPO accumulation. Furthermore, it has also been observed that the SCP2 activity inhibitor ScpI2 could not completely eliminate the damage of mitochondria and lysosomes by LPO after RSL3 treatment. Thus, other lipid carriers related to ferroptosis such as fatty acid binding protein (FABP) or oxysterol-binding protein (OSBP) might be involved [39].

Application of Fer-1 or ScpI2 to intervene in human primary chondrocytes from the early-stage OA could significantly improve cell viability. As such, 5-week modified-Hulth models were employed to simulate early-stage OA (identified by Safranin O/fast green staining) in rats [40]. Besides discovering that the SCP2 inhibitor ScpI2 reduced ferroptosis markers such as iron, ACSL4 and MDA in rat OA cartilage, ScpI2 also relieved the symptoms of OA which was consistent with the results of cartilage anabolism genes expression in vitro study, suggesting that articular injection of ScpI2 suppressed ferroptosis in chondrocytes thereby alleviating the progression of OA.

In summary, our study suggested that SCP2 mediated membrane damage of mitochondria through transporting LPO to mitochondria and accelerated the spread of LPO in RSL3-treated chondrocytes, resulting in ferroptosis (Fig. 6H). It also supported SCP2 being a potential therapeutic target for early-stage osteoarthritis involving ferroptosis.

## Materials and methods

### Analysis of differentially expressed genes and proteins

In this study, 259 ferroptosis-related genes were obtained from a ferroptosis database (FerrDb; Zhounan.org; dataset: drivers, suppressor, markers). The original sequencing data from GSE114007 was downloaded from the Gene Expression Omnibus (GEO) database, and expression profiles data for eight normal and ten OA knee cartilage tissue samples were based on GPL11454 (Illumina HiSeq 2000, *Homo sapiens*). The “edgeR” package was used to identify ferroptosis-related differentially expressed genes (FRDEGs), while the cut-off criteria were |log2 fold change (FC) | > 0.5 and a false discovery rate (FDR) < 0.05.

TMT-based quantitative proteomics was applied to identify the differentially expressed proteins from synovial fluid obtained from rabbit joints with or without articular cartilage injury as described in our previous work [41]. The proteins with *P* < 0.05 as well as |log2 fold change (FC) | > 0.5 were considered to be differentially expressed proteins.

### Ethics

This study was compliant with the Code of Ethics of the World Medical Association (Declaration of Helsinki) for experiments involving humans and relevant ethical regulations. Patients involved in the study provided informed consent, and this study was approved by the Ethical Committee of Guangzhou Red Cross Hospital of Jinan University (2021-187-01). All animal experiments were approved by the Animal Care and Use Committee of Guangzhou Red Cross Hospital of Jinan University (2021-191-01).

### Human samples

Human cartilage tissues with OA were obtained from five patients undergoing total knee replacement (TKR) surgery (designed as OA). Cartilage tissues without OA were obtained from three patients undergoing knee amputation (designed as Normal). Each cartilage specimen was used for both histological analysis and human primary chondrocytes extraction.

### OA rat model establishment

An OA model was established based on the modified-Hulth technique [40]. A total of 16 Sprague-Dawley rats (6 weeks old, female) were purchased from Guangdong Medical Laboratory Animal Center (Foshan, China) and randomly divided into four groups (*n* = 4): Sham, Hulth, Hulth + ScpI2 (0.1 mg/kg) (Vitas-M, Apeldoorn, Netherlands), Hulth + ScpI2 (0.5 mg/kg). One week after surgery, the rats were intraarticularly injected with vehicle or ScpI2 twice a week for 4 consecutive weeks. Then, 5 weeks after surgery, the rats were euthanized, and the knee joints were collected for further experiments.

### Isolation, culture of human or rat primary chondrocytes

Human or rat primary chondrocytes were isolated from articular cartilage fragments, which were dissected from femoral condyles and tibial plateau of patients or 3-week-old Sprague-Dawley rats. Human primary chondrocytes were used for lipid peroxidation and malondialdehyde detection. Rat chondrocytes were used for up to five generations in vitro experiments. Details in supplemental information.

### Western blotting

Rat chondrocytes were seeded in six-well plates at a density of 5 × 10^5^ cells per well. At 80% confluency, cells were treated with RSL3 (Sigma, MO, USA), SCP2 inducer 4-hydroxy-tamoxifen (4OHT; Macklin, Shanghai, China) or SCP2 specific activity inhibitor ScpI2. Then, Western blotting was performed to analyze SCP2 expression. The membranes were incubated overnight with SCP2 antibody (1:500; Proteintech, Wuhan, China), cytochrome C antibody (1:500; R&D Systems, MN, USA), β-actin antibody (1:5000; R&D Systems, MN, USA), or GAPDH antibody (1:1,000; Arigo, Taiwan, China) at 4 °C and then incubated with secondary antibodies (1:5,000; Sigma, MO, USA) for 1 h at room temperature. The reacted proteins were detected and visualized using SuperSignal West Pico Chemiluminescent Substrate (Thermo Scientific, CA, USA), and images were obtained with a Bio-Rad scanner.

### Cell viability assay

Cell viability was measured by Cell Counting Kit-8 (Beyotime, Shanghai, China). Human primary chondrocytes from OA cartilage were treated with ferroptosis inhibitor Fer-1 or SCP2 activity inhibitor ScpI2. Additionally, rat chondrocytes were treated with ferroptosis inducer RSL3 or in combination with SCP2 inducer 4OHT or inhibitor ScpI2. Subsequently, the cells were exposed to 100 μl of a 10% CCK-8 solution for 1 h at 37 °C, 5% CO_2_ in an incubator. The absorbance at 450 nm was measured using a microplate reader (GloMax Multi Plus, Promega).

### Determination of intracellular iron levels

Rat chondrocytes were seeded onto 6-well plates (2 × 10^5^ cells per well). On the following day, cells were treated with RSL3 alone or in combination with 4OHT or ScpI2. The cells were then harvested and counted. The total iron content of the cells in each group was analyzed using an Iron Assay Kit according to the manufacturer’s protocol (Sigma, MO, USA) on a colorimetric microplate reader (the absorbance is 600 nm).

### Malondialdehyde (MDA) assay

Rat chondrocytes (RSL3, 4OHT or ScpI2 treated) or human primary chondrocytes were lysed and rat cartilage tissue was homogenized. The MDA concentration was measured by Lipid Peroxidation MDA Assay Kit (Beyotime, Shanghai, China) according to the manufacturer’s instructions at the absorbance of 560 nm. The protein concentrations were quantified using a BCA Protein Assay Kit to normalize the MDA content.

### Lipid peroxidation and reactive oxygen species (ROS) assay

For lipid peroxidation assay, rat chondrocytes (RSL3, 4OHT or ScpI2 treated) or human primary chondrocytes were stained with 10 μM BODIPY 581/591 C11 (Invitrogen, CA, USA) at 37 °C for 30 min. For ROS assay, the treated rat chondrocytes were stained with 10 μM DCFH-DA (Beyotime, Shanghai, China) at 37 °C for 20 min. After incubation, the cells were washed with PBS and harvested by trypsinization, and resuspended in 200 μL PBS. Lipid peroxidation and ROS were assessed using the flow cytometer BD FACSVerse (Becton Dickinson) with the FITC filter. A minimum of 10,000 cells were analyzed for each sample. Data analysis was performed using the FlowJo v10 (BD Bioscience).

### Adenosine triphosphate (ATP) assay

ATP level was measured using an Enhanced ATP Assay Kit (Beyotime, Shanghai, China) according to the manufacturer’s protocol.

### Mitochondrial membrane potential (MMP) detection

MMP of chondrocytes was measured by fluorescence microscopy using Mitochondrial Membrane Potential Assay Kit with JC-1 staining (Beyotime, Shanghai, China) according to the manufacturer’s protocol.

### Lysosomal membrane permeabilization (LMP) and lysosome staining

LMP could be measured by acridine orange (Bjbalb, Beijing, China) staining and lysosomes could be visualized by Lyso-Tracker Red (Beyotime, Shanghai, China) with a fluorescence microscopy.

### Lactate dehydrogenase (LDH) release assay

Cell membrane integrity could be estimated by LDH concentration in the supernatant. LDH Assay Kit (Beyotime, Shanghai, China) was employed to determine the LDH release following the manufacturer’s instructions.

### Trypan blue staining

Trypan blue (Beyotime, Shanghai, China) reflects the rupture of the cell membrane. The number of blue cells in each well was determined by a microscope and the percentage of positive cells was calculated.

### Colocalization of Lipid hydroperoxides (LPO) with mitochondria or lysosomes

The colocalization of LPO with organelles was measured using BODIPY 665/676 (Invitrogen, CA, USA) to track LPO and CellLight-GFP-BacMam-2.0 (Invitrogen, CA, USA) to track mitochondria or lysosomes in rat chondrocytes. Briefly, rat chondrocytes were seeded in a 24-well plate and cultured overnight. After being transfected with CellLight-GFP-BacMam-2.0 (2 μL of BacMam reagent per 10,000 cells) for 24 h, the cells were treated with RSL3, 4OHT, ScpI2, colchicine (Macklin, Shanghai, China), DIDS (MCE, NJ, USA), CCCP (MCE, NJ, USA), Mitoquinol (MitoQ, Glpbio, CA, USA) or chloroquine (CQ; Macklin, Shanghai, China). BODIPY 665/676 (20 μM, 30 min) and Hoechst 33258 (10 μg/mL, 10 min) were used for further staining. Images were obtained by a fluorescence microscope (Nikon, Eclipse-Ti) : fluorescence excitation/emission 561⁄600 nm for Oxidized BODIPY, 458⁄520 nm for GFP, and 352⁄461 nm for Hoechst.

### Immunofluorescence staining and subcellular localization of SCP2

Rat chondrocytes were seeded in a 24-well plate and cultured overnight. After being transfected with CellLight-GFP-BacMam-2.0 (2 μL / 10,000 cells) for 24 h to track mitochondria or lysosomes, the cells were treated with RSL3, 4OHT, ScpI2, DIDS, CCCP, CQ or colchicine. The cells were then fixed in 4% paraformaldehyde (Beyotime, Shanghai, China), permeabilized with 0.2% Triton-X (Beyotime, Shanghai, China), and blocked with 2% BSA (Sigma, MO, USA) for 0.5 h at room temperature. After overnight incubation with SCP2 antibody (1:100) at 4 °C, the cells were incubated with a Cy3-conjugated secondary antibody (1:100; Servicebio, Wuhan, China) at room temperature for 1 h followed by Hoechst 33258 (10 μg/mL) for 5 min. Images were acquired using a fluorescence microscope (Nikon, Eclipse-Ti) : fluorescence excitation/emission 550⁄570 nm for SCP2, 458⁄520 nm for GFP, and 352⁄461 nm for Hoechst.

### Detection of mitochondrial lipid peroxidation and mitochondrial ROS

Mitochondrial lipid peroxidation and mitochondrial ROS were measured by MitoPeDPP (Dojindo, Kumamoto, Japan) and MitoSox (Invitrogen, CA, USA) staining, respectively.

### Mitochondria isolation and mitochondrial protein extraction

Mitochondria were extracted using a Mitochondria Isolation Kit (Beyotime, Shanghai, China). The content of SCP2 and cytochrome C in mitochondria was analyzed by Western blotting, with VDAC antibody (1:1000; Proteintech, Wuhan, China) as mitochondrial internal reference.

### Determination of the binding rate of SCP2 and 15(S)–HpETE

The binding rate of SCP2 and 15(S)–HpETE (a monohydroperoxy polyunsaturated fatty acid in cells) was determined by a Fast Micro-Equilibrium Dialyzer (Harvard Apparatus) and LC-MS/MS (AB sciex, API 4000 Q-Trap). Recombinant SCP2 (5 μM; Sangon, Shanghai, China) and 15(S)–HpETE (5 μM; Glpbio, CA, USA) were mixed with or without ScpI2 (5 μM) in phosphate buffered saline (PBS; Corning, NY, USA) and then filled in one chamber. The other chamber was filled with blank PBS. A 3.5 kDa dialysis membrane (Viskase, USA) was applied to separate the chambers which were dialyzed for 2 h at 37 °C in a shaker. A mixture of SCP2 (5 μM) and GSH (5 μM; Macklin, Shanghai, China) served as negative control. After dialysis, the solution from both sides was subjected to compound extraction and LC-MS/MS analysis.

### Histology and immunohistochemical assay

The primary antibodies used for immunohistochemistry staining were COL2A1 (1:100; Servicebio, Wuhan, China), MMP13 (1:100; Servicebio, Wuhan, China), SCP2 (1:50; Zen-bio, Chengdu, China), GPX4 (1:50; Chengdu, China), or ACSL4 (1:20; Proteintech, Wuhan, China). The image was captured by microscope (Nikon, Eclipse Ci) and the number of positive cells/total cells per field in each group were counted under 200-time magnification. The progression of OA was evaluated by the Osteoarthritis Research Society International (OARSI) scoring system in a blind manner.

### Statistical analysis

Bars and error bars are presented as the mean ± standard deviation (SD) of at least three independent replicates. Statistical analyses were performed using GraphPad Prism 9.0.0 (GraphPad Software). Statistically significant differences were evaluated using unpaired two-tailed Student’s *t* tests for comparison between two groups or by one-way analysis of variance followed by a Bonferroni post hoctest to determine differences among groups of more than two. *A P* value less than 0.05 was considered significant.

## Supplementary information

Supplementary material

Full length uncropped original western blots

## Data Availability

The data that support the findings of this study are available from the corresponding authors on reasonable request.
