# Supplementary material for: SCP2 mediates the transport of lipid hydroperoxides to mitochondria in chondrocyte ferroptosis
Source: Cell Death Discov. 2023 Jul 8;9:234. doi: 10.1038/s41420-023-01522-x (PMC10329676; doi:10.1038/s41420-023-01522-x)
Supplement: Supplementary file 1 — Supplementary material [file 41420_2023_1522_MOESM1_ESM.docx]

**Supplementary material
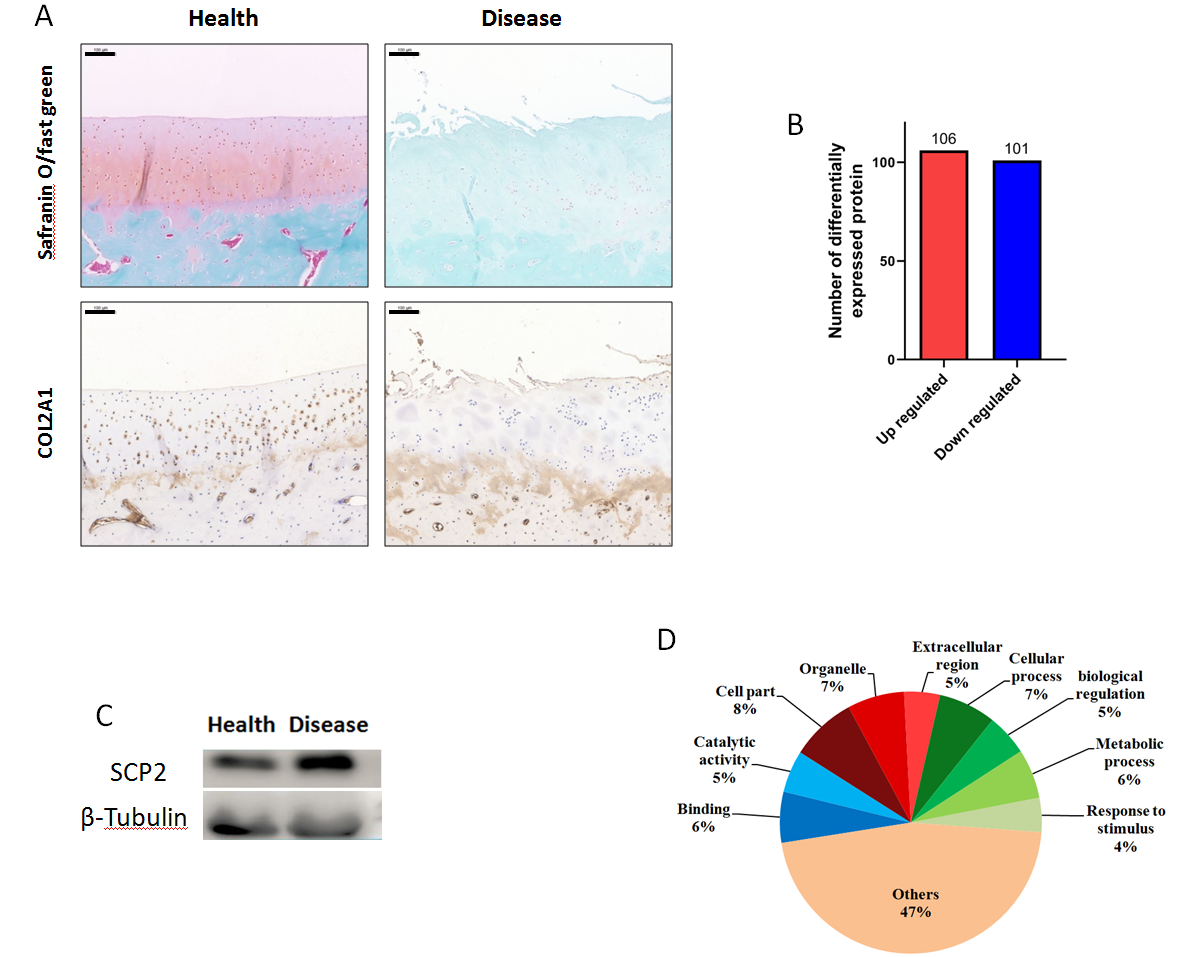
**

**Fig. S1. Proteomics analysis of the differentially expressed proteins from rabbit joints.** (**A**) Safranin O/fast green and immunohistochemistry staining of COL2A1 in articular cartilage of rabbits at 30 weeks after surgery (n = 3 per group). (**B**) The number of differentially expressed proteins in the synovial fluid from rabbit joints between injured (n = 3 per group) and normal (n = 3 per group) cartilage at 12 weeks after surgery. (**C**) Western blot of SCP2 in the synovial fluid from rabbit joints at 12 weeks after surgery (n = 3 per group). (**D**) Gene ontology annotation of the differentially expressed proteins identified in the synovial fluid from rabbit joints.

**Fig. S2. Studies on the cell viability after indicated treatments and the binding rate of SCP2 and 15(S)-HpETE.** (**A**) Cell viability of human primary chondrocytes from OA cartilage was quantified by CCK-8 post indicated treatment: Fer-1 (5 μM) or ScpI2 (5 μM) (n = 3 per group). (**B**) Western blot of GPX4 in rat chondrocytes after indicated treatment: RSL3 (0.25 μM) or ScpI2 (5 μM) for 4 h (n = 3 per group). (**C**) Cell viability of rat chondrocytes was quantified by CCK-8 treated with different concentrations of 4OHT (n = 3 per group). (**D**) Cell viability of rat chondrocytes was quantified by CCK-8 treated with different concentrations of ScpI2 (n = 3 per group). Data are expressed as means+SD. Unpaired two-tailed t-tests,**P* < 0.05.

**Fig. S3. Colocalization of mitochondria with LPO or SCP2 under different treatments.** (**A**) The images of BODIPY665/676, CellLight-mito-GFP, and Hoechst33258 staining in rat chondrocytes treated with RSL3 (0.25 μM), ScpI2 (5 μM), MitoQ (1 μM), colchicine (5 nM), DIDS (200 μM) or CCCP (100 μM). Scale bar, 50μm. (**B**) The images of immunofluorescence SCP2, CellLight-mito-GFP, and Hoechst33258 staining in rat chondrocytes treated with RSL3 (0.25 μM), colchicine (5 nM), DIDS (200 μM) or CCCP (100 μM). Scale bar, 50μm. (**C**) Mitochondrial membrane potential of rat chondrocytes treated with CCCP (100 μM) detected by JC-1 staining. Scale bar, 100μm. (**D**) The relative colocalization coefficients of mitochondria and LPO with mito-GFP and BODIPY staining (n = 6 per group). (**E**) The relative colocalization coefficients of mitochondria and SCP2 with mito-GFP and immunofluorescence staining (n = 6 per group). Data are expressed as means+SD. Unpaired two-tailed t-tests,* *P*< 0.05.

**Fig. S4.** Representative photographs of rat articular cartilage at 5 weeks after surgery taken by a stereoscopic microscope (Nikon SMZ18). Red arrows point out the medial side of the joint. Green arrows indicate the cartilage surface that was rough and thin. Compared with the Sham group, the cartilage in the Hulth group showed obvious wear. The application of ScpI2 alleviated cartilage injury, especially in the 0.5mg/kg group.

**Tables S1**. Ferroptosis-related differentially expressed genes in OA cartilage.

| **id** | **logFC** | **logCPM** | **PValue** | **FDR** |
| --- | --- | --- | --- | --- |
| RGS4 | 3.00863 | 6.012304 | 0.000432 | 0.002468 |
| RRM2 | 2.869881 | 6.734791 | 0.001261 | 0.005995 |
| AKR1C2 | 2.457744 | 11.93861 | 1.52E-07 | 2.72E-06 |
| ALOX5 | 1.787113 | 5.985815 | 0.009785 | 0.034329 |
| AURKA | 1.607093 | 6.261923 | 0.008331 | 0.030218 |
| TF | 1.596971 | 11.21892 | 0.00213 | 0.009498 |
| DPP4 | 1.575616 | 8.634756 | 0.003003 | 0.012358 |
| PRDX1 | 1.565258 | 12.69365 | 6.81E-05 | 0.000486 |
| ENPP2 | 1.559982 | 9.34245 | 1.38E-06 | 1.74E-05 |
| SLC2A12 | 1.542115 | 9.384212 | 9.72E-05 | 0.00065 |
| NQO1 | 1.539081 | 8.528136 | 2.37E-05 | 0.00022 |
| SLC40A1 | 1.456675 | 10.98863 | 0.000875 | 0.004638 |
| STEAP3 | 1.404456 | 12.95128 | 5.88E-05 | 0.00045 |
| ATG16L1 | 1.157183 | 8.939684 | 5.25E-05 | 0.00043 |
| SCP2 | 1.130071 | 11.94363 | 0.002923 | 0.012264 |
| RIPK1 | 1.107742 | 10.01987 | 0.000208 | 0.001236 |
| WIPI1 | 1.102127 | 10.72927 | 5.42E-05 | 0.00043 |
| NRAS | 0.984603 | 9.671517 | 0.001113 | 0.005415 |
| CD44 | 0.91827 | 13.80212 | 0.004239 | 0.016495 |
| TGFBR1 | 0.906624 | 13.01162 | 0.010122 | 0.034382 |
| EIF2AK4 | 0.839033 | 9.83177 | 0.003873 | 0.015638 |
| LAMP2 | 0.816256 | 12.08516 | 0.001833 | 0.008527 |
| MAPK9 | 0.801443 | 8.496428 | 0.006551 | 0.024595 |
| KRAS | 0.799172 | 9.667279 | 0.002843 | 0.012166 |
| NCOA4 | 0.65641 | 11.65213 | 0.006488 | 0.024595 |
| YY1AP1 | -0.83212 | 11.10052 | 0.004184 | 0.016495 |
| PLIN2 | -0.88397 | 9.315476 | 0.000889 | 0.004638 |
| GPX4 | -0.96505 | 11.11239 | 0.010103 | 0.034382 |
| ACSF2 | -0.97782 | 10.87384 | 0.011608 | 0.038816 |
| EGFR | -1.05947 | 10.76811 | 0.002247 | 0.009814 |
| LONP1 | -1.08041 | 11.09005 | 2.54E-05 | 0.000226 |
| ARRDC3 | -1.09225 | 12.33805 | 0.00889 | 0.031708 |
| PRKAA2 | -1.10474 | 9.279679 | 6.39E-05 | 0.000471 |
| SLC2A3 | -1.15098 | 10.96503 | 0.001963 | 0.008936 |
| LPIN1 | -1.1523 | 11.41686 | 0.000962 | 0.0049 |
| PCK2 | -1.15514 | 9.695566 | 4.76E-05 | 0.000407 |
| PTGS2 | -1.15884 | 9.267872 | 0.013023 | 0.042874 |
| ACSL4 | -1.25257 | 12.02621 | 0.006747 | 0.024895 |
| RELA | -1.30995 | 12.31516 | 0.000172 | 0.001053 |

Table S1 (continued).

| VLDLR | -1.41803 | 9.160691 | 0.000158 | 0.000996 |
| --- | --- | --- | --- | --- |
| GABARAPL1 | -1.39243 | 11.72807 | 0.000438 | 0.002468 |
| HERPUD1 | -1.41303 | 13.46867 | 7.80E-07 | 1.04E-05 |
| CHAC1 | -1.4327 | 7.239528 | 0.001076 | 0.005354 |
|  |  |  |  |  |
| ASNS | -1.47077 | 9.792615 | 1.18E-05 | 0.000115 |
| CEBPG | -1.49027 | 10.25273 | 5.00E-10 | 1.34E-08 |
| SLC7A5 | -1.51545 | 10.04117 | 0.000129 | 0.000839 |
| UBC | -1.55822 | 15.95262 | 5.65E-08 | 1.10E-06 |
| SLC3A2 | -1.68942 | 11.35599 | 7.47E-07 | 1.04E-05 |
| ZFP36 | -1.74013 | 13.70981 | 5.10E-06 | 5.46E-05 |
| ANGPTL7 | -1.74573 | 10.80147 | 0.000774 | 0.004246 |
| ULK1 | -1.78583 | 10.57213 | 1.75E-07 | 2.89E-06 |
| SLC2A1 | -1.95426 | 12.90681 | 2.42E-07 | 3.70E-06 |
| GPT2 | -1.96554 | 11.35477 | 1.71E-11 | 6.23E-10 |
| JUN | -2.0801 | 12.35622 | 2.98E-06 | 3.36E-05 |
| BNIP3 | -2.09941 | 14.23396 | 1.05E-09 | 2.49E-08 |
| ARNTL | -2.1306 | 9.61278 | 5.16E-08 | 1.10E-06 |
| TXNIP | -2.26944 | 14.7265 | 2.38E-06 | 2.83E-05 |
| ATF3 | -2.27418 | 8.916423 | 7.07E-06 | 7.21E-05 |
| DDIT3 | -2.42123 | 10.72171 | 3.19E-20 | 6.82E-18 |
| TSC22D3 | -2.43326 | 14.27739 | 8.09E-05 | 0.000559 |
| VEGFA | -2.61109 | 15.81271 | 1.66E-11 | 6.23E-10 |
| SESN2 | -2.69267 | 9.560173 | 1.89E-13 | 2.03E-11 |
| CDKN1A | -2.70487 | 10.95643 | 4.24E-10 | 1.29E-08 |
| DDIT4 | -3.21386 | 15.45198 | 1.75E-11 | 6.23E-10 |
| HILPDA | -3.30893 | 13.20255 | 4.78E-13 | 3.41E-11 |

**Table S2.** Information of patients.

| **No.** | **Gender/Age** | **Cartilage site** | **Operation** |
| --- | --- | --- | --- |
| 1 | Female/66 | Lateral femoral condyle | Total knee replacement |
| 2 | Female/58 | Lateral femoral condyle | Total knee replacement |
| 3 | Female/81 | Lateral femoral condyle | Total knee replacement |
| 4 | Male/83 | Lateral tibial plateau | Total knee replacement |
| 5 | Male/66 | Lateral femoral condyle | Total knee replacement |
| 6 | Male/64 | Lateral tibial plateau | Knee amputation |
| 7 | Male/57 | Lateral tibial plateau | Knee amputation |
| 8 | Female/19 | Lateral femoral condyle | Knee amputation |


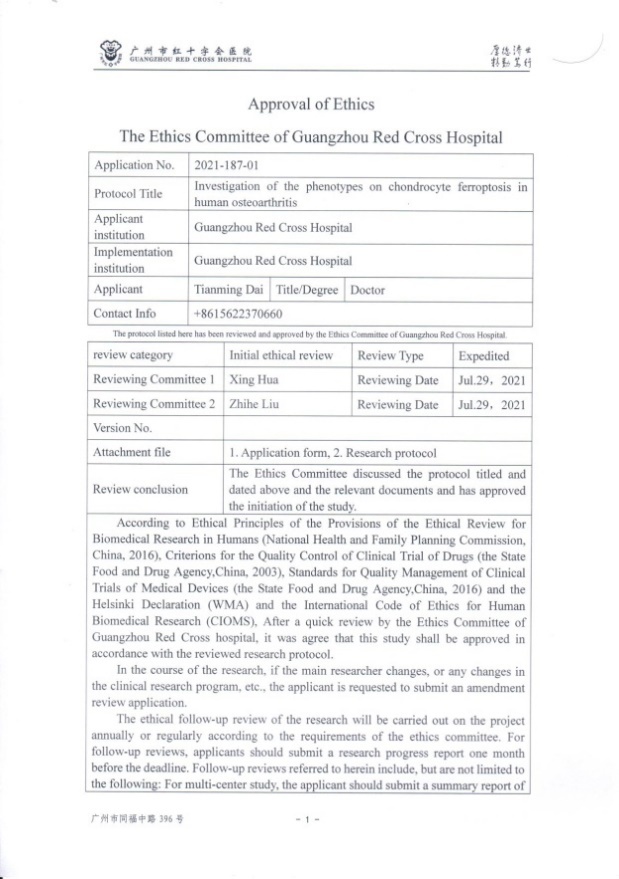

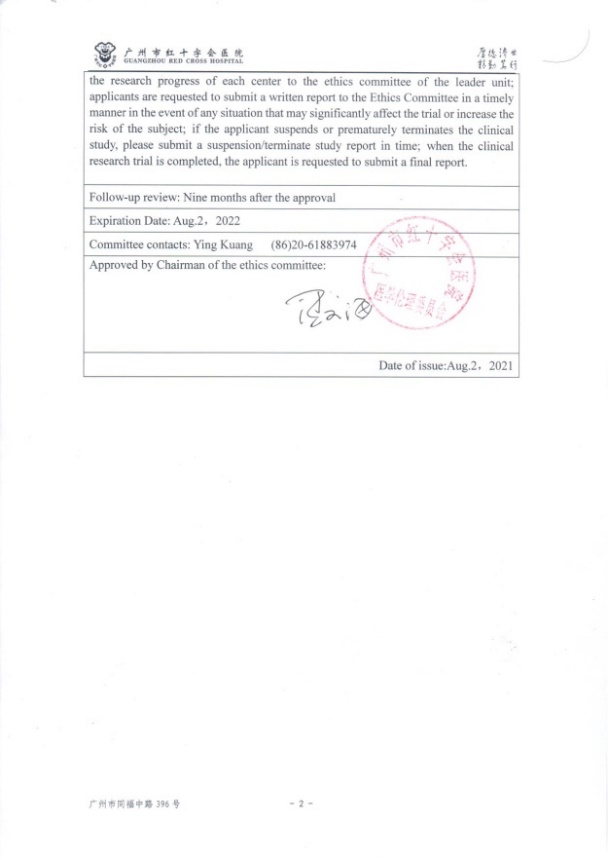


Approval of Ethics in clinical research.


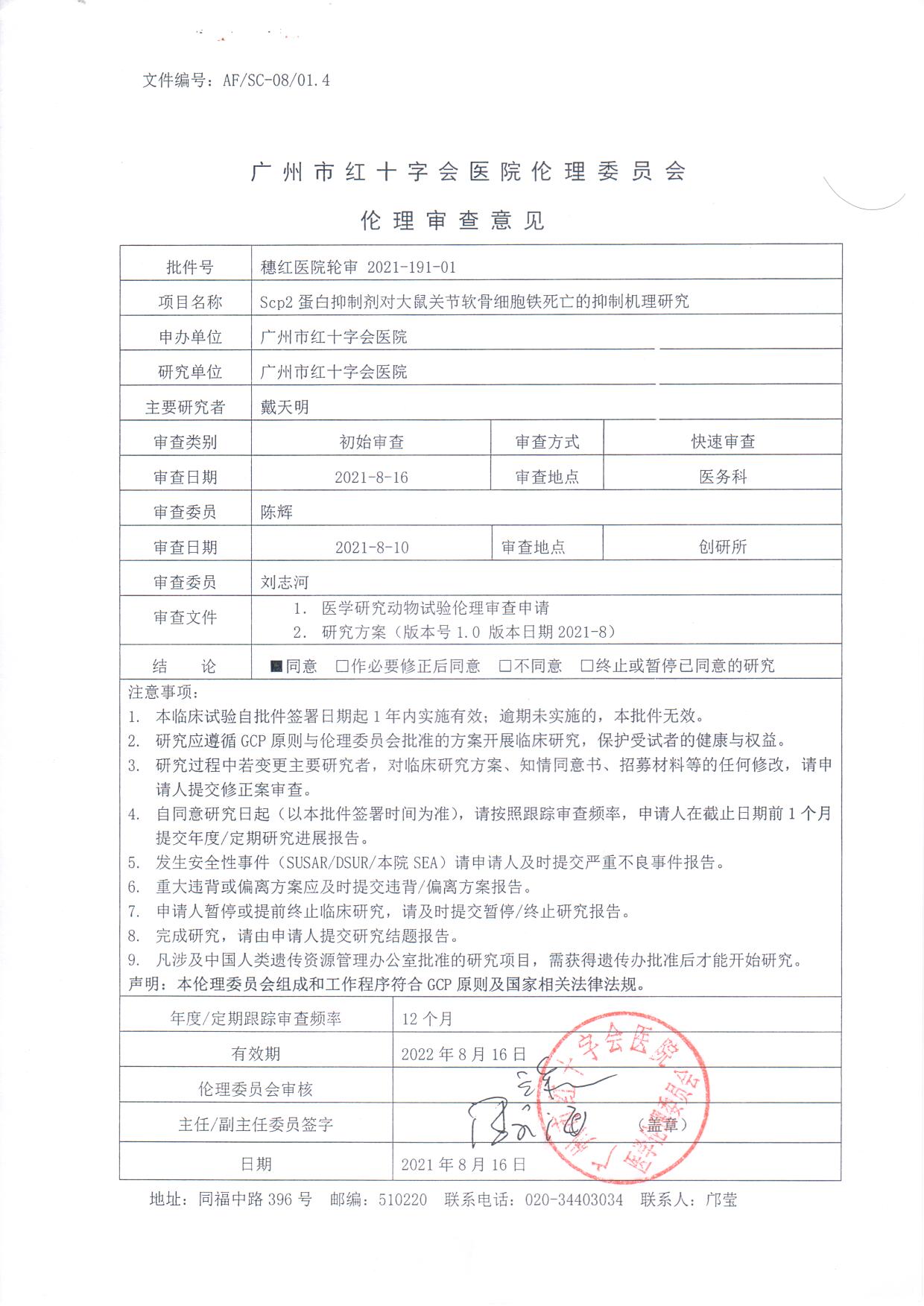


Approval of Ethics in animal research.

**Supplementary Materials and Methods**

**OA rat model establishment**

A total of 16 Sprague-Dawley rats (6 weeks old, female) were weighed and numbered, randomly divided into four groups (n=4) according to a random-number table.

After anesthesia by intraperitoneal injection of 30 mg/kg sodium pentobarbital, transection of the anterior cruciate ligament and resection of the medial meniscus of the right knee joint were performed in the 3 Hulth groups. The Sham group only received a skin incision and suturing. Animal death caused by anesthesia or surgical risks would be excluded.

**Isolation, culture of** **human or rat** **primary chondrocytes**

Briefly, cartilage fragments were firstly digested with 0.25% trypsin (Gibco, CA, USA) at 37°C for 30 min and then fully digested using 0.2% collagenase II (Sigma,MO, USA) at 37°C for 6 h. After dispensing the digested cartilage fragments through a 70-μm cell strainer, primary chondrocytes were cultured with Dulbecco’s modified Eagle’s medium - F12 (DMEM/F12; Gibco, Hangzhou, China) supplemented with 10% fetal bovine serum (Gibco, CA, USA), 1% penicillin/streptomycin cocktail (Gibco,CA, USA) under standard conditions (37 °C, 5% CO2) for 5 to 10 days.

**Western blotting**

Briefly, cells from different treatment groups were harvested with CelLytic M Reagent (Sigma, MO, USA) containing 1% protease Inhibitor Cocktail (Sigma,MO, USA) for 40 min on ice. The extract was collected and centrifuged at 12,000 × g and 4°C for 30 min. Then, the supernatant was collected, and the protein concentration of each sample was detected with a BCA Protein Assay Kit (Beyotime, Shanghai, China). Samples containing a quantity of proteins were separated by sodium dodecyl sulfate–polyacrylamide gel electrophoresis (SDS-PAGE) and transferred to PVDF membranes (Sigma, MO, USA). After blocking with 5% skim milk at room temperature for 1 h, the membranes were incubated overnight with antibody.

SCP2 antibody (1:500; Proteintech, 23006-1-AP, Wuhan, China);

cytochrome C antibody (1:500; R&D Systems, MAB897, MN, USA);

β-actin antibody (1:5000; R&D Systems, MAB8929, MN, USA);

GAPDH antibody (1:1,000; Arigo, ARG10112, Taiwan, China)

**Cell viability assay**

Rat chondrocytes were seeded onto 96-well plates (5,000 cells per well).The next day, the cells were treated with ferroptosis inducer RSL3 at a series of concentrations alone or in combination with SCP2 inducer 4OHT or inhibitor ScpI2.

Human OA primary chondrocytes were isolated from early OA cartilage in the lateral tibial plateau and lateral condyle. The cells were directly treated with ferroptosis inhibitor Fer-1 (MCE, NJ, USA) or SCP2 inhibitor ScpI2 without RSL3 treatment.

**RNA extraction and quantitative real-time polymerase chain reaction (qRT-PCR)**

RNA was extracted using Trizol (Invitrogen), and total RNA was reverse transcribed into cDNA using a reverse transcription kit (Takara, China). Real-time quantitative polymerase chain reactions were performed with the SYBR Green RT-PCR reagent (Takara). Gene expression levels were normalized to GAPDH using the 2^−ΔΔCt^ method. These primer sequences are listed in Table 1.

Table 1. Sequence information for primers (5’ to 3’).

| Gene | Forward Primer 5’-3’ | Reverse Primer 5’-3’ |
| --- | --- | --- |
| COL2A1 | GAGTGGAAGAGCGGAGACTACTG | CTCCATGTTGCAGAAGACTTTCA |
| ACAN | CTAGCTGCTTAGCAGGGATAACG | TGACCCGCAGAGTCACAAAG |
| ADAMTS4 | ACCCTCCGAACGACTCAGAT | ATACCCAGAGCGTCACAGGT |
| MMP13 | TTTGAGGACACGGGGAAGAC | GCGGGGATAGTCTTTGTCCAT |

**Adenosine triphosphate (ATP) assay**

Briefly, rat chondrocytes were seeded onto 6-well plates (2 × 105 cells per well) with indicated treatment and then lysed in an ATP lysis buffer. The ATP level was determined using the luminometer function on a colorimetric microplate reader. The protein concentrations were quantified using a BCA Protein Assay Kit to normalize the ATP level.

**Mitochondrial membrane potential (MMP) detection**

Briefly, rat chondrocytes were seeded in a 24-well plate with indicated treatment. Subsequently, the cells were incubated with JC-1 staining solution for 20 min at 37 °C and imaged with a fluorescence microscope (Nikon, Eclipse-Ti) on fluorescence excitation/emission maxima: 514/529 nm, monomer form; 585/590 nm J-aggregate form.

**Lysosomal membrane permeabilization (LMP) and lysosome staining**

Briefly, rat chondrocytes were seeded in a 24-well plate with indicated treatment. For acridine orange staining, the cells were incubated with 10 μg/mL acridine orange at 37 °C for 10 min and then washed with DMEM/F12. For Lyso-Tracker Red staining, cells were incubated with 0.1 μM Lyso-Tracker Red for 40 min followed by 10 μg/mL Hoechst 33258 (Beyotime, Shanghai, China) for 10 min at 37 °C. The cells were imaged with a fluorescence microscope (Nikon, Eclipse-Ti) : fluorescence excitation/emission 490⁄526 nm and 555⁄617 nm for acridine orange, 577⁄590 nm for Lyso-Tracker Red, and 352⁄461 nm for Hoechst.

**Lactate dehydrogenase (LDH) release assay**

Briefly, rat chondrocytes were seeded in a 96-well plate and cultured overnight. After indicated treatment, the supernatant of each well was mixed with 60 µL work solution and maintained at room temperature for 30 min in the dark. The mixed solution was detected at 490 nm of absorbance using a colorimetric microplate reader.

**Trypan blue staining**

Approximately, rat chondrocytes were seeded in a 48-well plate with indicated treatment. Next, 100 μL trypan blue staining solution was added to each well for 5 min and then washed three times with PBS.

**Colocalization of mitochondria and lysosomes**

Colocalization of mitochondria and lysosomes was measured using Mito-tracker green (Beyotime, Shanghai, China) or Lyso-tracker red (Beyotime, Shanghai, China) to track mitochondria or lysosomes in rat chondrocytes after RSL3, 4OHT, ScpI2, CQ treatment.

**Detection of mitochondrial lipid peroxidation and mitochondrial ROS**

Briefly, overnight plated rat chondrocytes on 24-well plates were treated with RSL3, 4OHT, ScpI2, colchicine, or Mito-Q (Glpbio, CA, USA). The cells were simultaneously incubated with 3 μM MitoSox and 10 μg/mL Hoechst 33258 for 10 min followed by 1 μM MitoPeDPP for 15 min at 37 °C. After being washed with DMEM/F12, the cells were imaged with a fluorescence microscope (Nikon, Eclipse-Ti) : fluorescence excitation/emission 470⁄525 nm for Oxidized-MitoPeDPP, 510⁄580 nm for MitoSox, and 352⁄461 nm for Hoechst.

**Mitochondria isolation and mitochondrial protein extraction**

Briefly, chondrocytes were collected and then incubated with cold mitochondrial isolation reagent for 15 min. Cells were homogenized and centrifuged at 1,000 g for 10 min. The supernatant was then centrifuged at 11,000 g for 10 min. The sediment was blended with mitochondrial lysate solution to obtain mitochondrial proteins.

**The LC-MS/MS method fordetermination of the binding rate of SCP2 and** **15(S)–HpETE**

For free 15(S)–HpETE or GSH quantification, an aliquot of dialysis solution (100 μL) was extracted with 300 μL methanol containing 0.2 μg/mL apigenin as an internal standard. 15(S)–HpETE or GSH was analyzed by a WelchXB-C18 (2.1×50 mm, 3.0 µm, Welch) at room temperature with a flow rate of 0.4 mL/min. The mobile phase consisted of methanol and water with 5mM ammonium formate running a gradient profile. Compounds were quantified by negative ionization conditions including source temperature, 500°C; ion spray voltage, -4500 V and curtain gas, 20 psi. The ion pairs for the multiple reaction monitoring were m/z 335 to 317 for 15(S)–HpETE, m/z 306 to 143 for GSH, and m/z 269 to 117 for apigenin. Calibration standards and quality control samples were run in parallel under the same conditions.

**Histology and immunohistochemical assay**

Fresh cartilage tissues from human or proximal tibia from rats were fixed in 4% buffered paraformaldehyde for 24 h. The proximal tibia of rats was first decalcified with 10% EDTA solution for 4 weeks. After being embedded in paraffin wax, the tissues were sectioned to 5 μm thickness and stained with Safranin O/fast green or stained with prussian blue (enhance with DAB).For immunohistochemistry staining, sections were deparaffinized, antigen retrieved and blocked. After incubated with primaryantibodies, sections were incubated with secondary antibody (Servicebio, Wuhan, China), followed by counterstained with hematoxylin and visualized by DAB (Servicebio, Wuhan, China).

COL2A1 (1:100; Servicebio, GB11021, Wuhan, China);

MMP13 (1:100; Servicebio, GB11247, Wuhan, China);

SCP2 (1:50; Zen-bio, 823100, Chengdu, China);

GPX4 (1:50; R381958, Chengdu, China);

ACSL4 (1:20; Proteintech, 22401-1-AP, Wuhan, China).
