# Supplementary material for: SCP2 mediates the transport of lipid hydroperoxides to mitochondria in chondrocyte ferroptosis
Source: Cell Death Discov. 2023 Jul 8;9:234. doi: 10.1038/s41420-023-01522-x (PMC10329676; doi:10.1038/s41420-023-01522-x)
Supplement: Supplementary file 2 — Full length uncropped original western blots [file 41420_2023_1522_MOESM2_ESM.pdf]

Full length uncropped original western blots.

Fig.2A SCP2-Cytosol

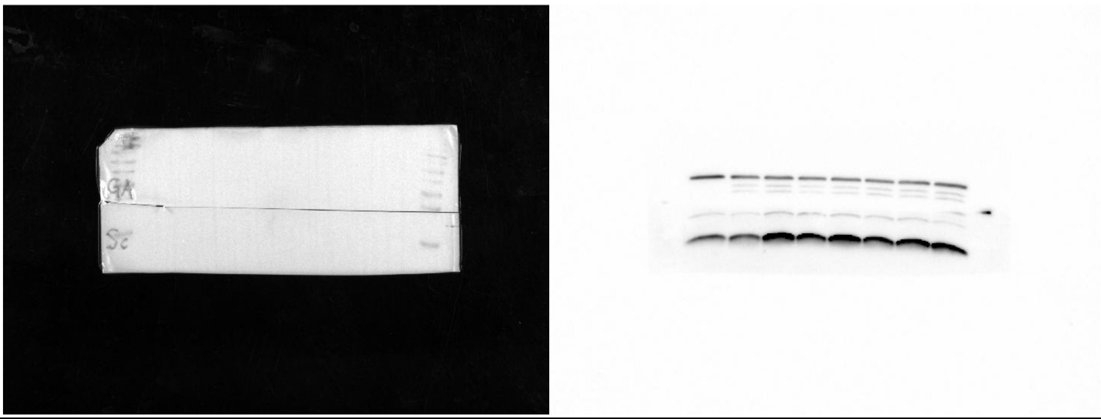

Marker	Control	RSL-3	4OHT	Scpi2	R+4	R+S	R+4+S	—	Marker

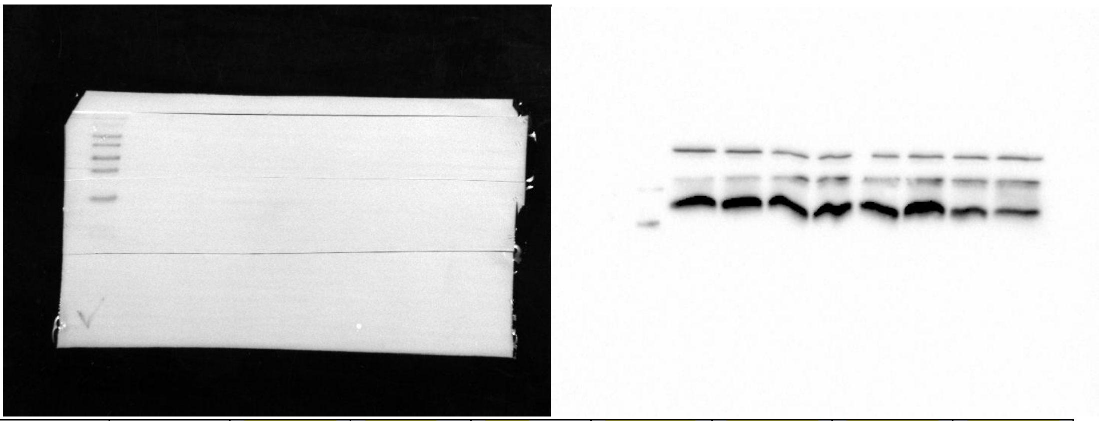

Marker	—	R+4+S	R+S	R+4	Scpi2	4OHT	RSL-3	Control

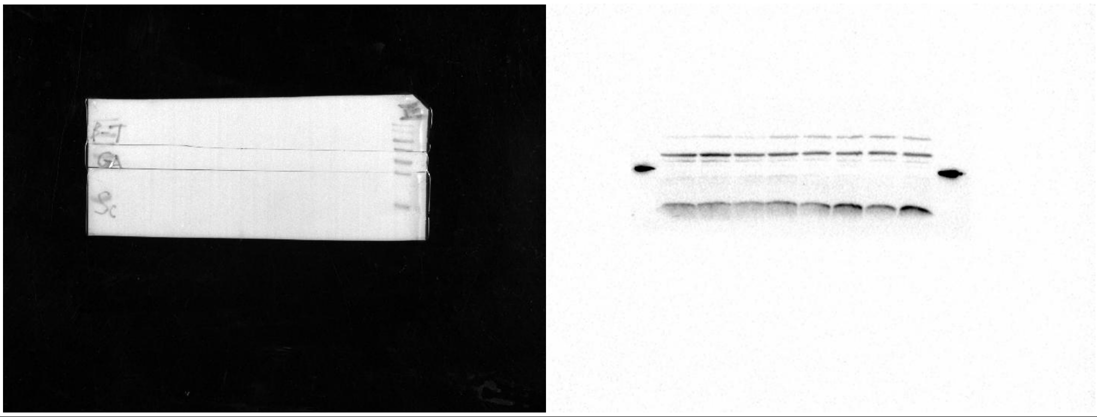

Marker	Control	R+4+S	R+S	R+4	Scpi2	4OHT	RSL-3	—	Marker

Fig.4E SCP2-Mitochondria

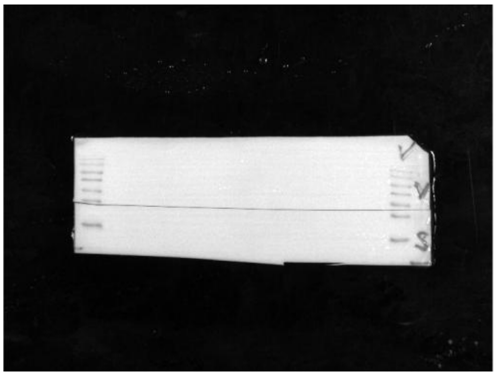

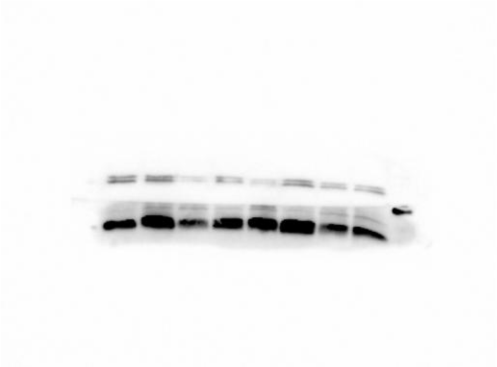

Marker	Control	RSL-3	R+4	R+S	Control	RSL-3	R+4	R+S	Marker

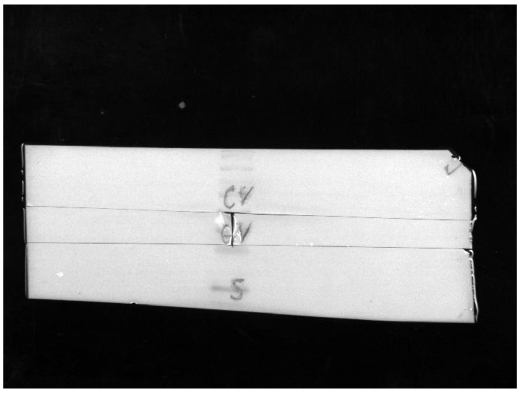

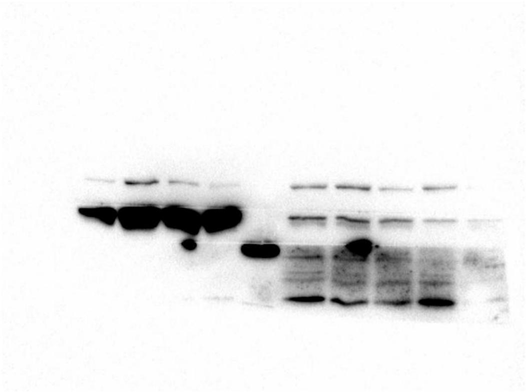

—	—	—	—	Marker	—	Control	RSL-3	R+4	R+S

Fig.3C CytC-Mitochondria

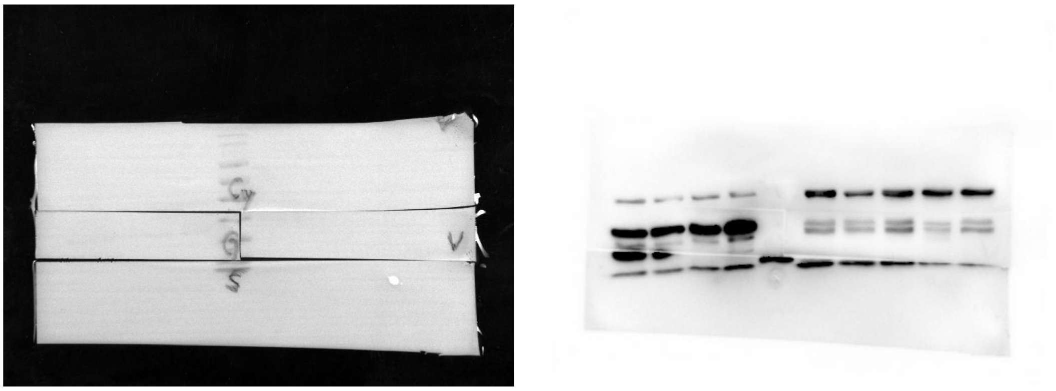

—	—	—	—	Marker	Control	RSL-3	R+4	R+S	—

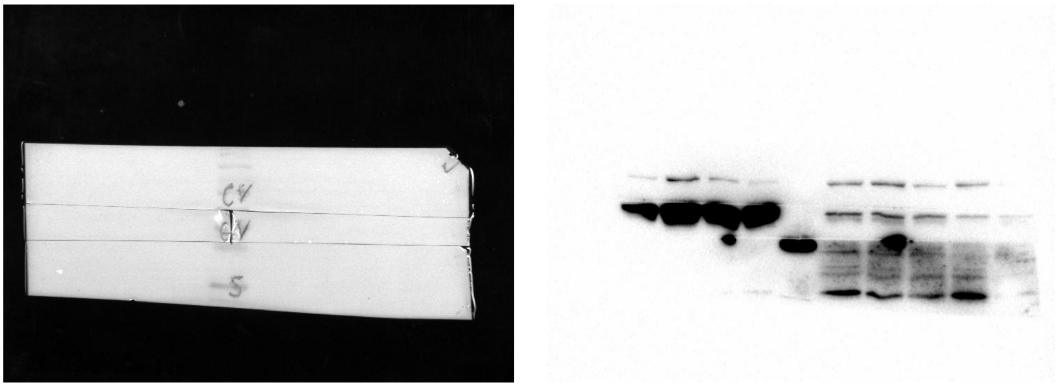

—	—	—	—	Marker	Control	RSL-3	R+4	R+S	—

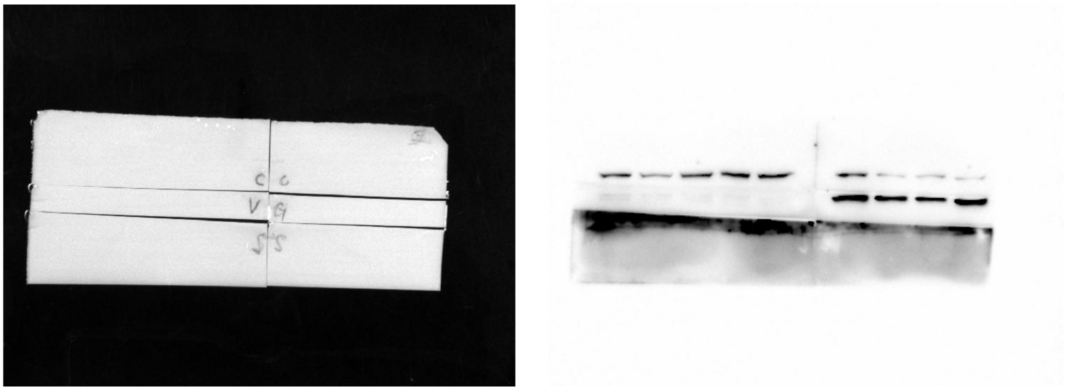

—	—	—	—	—	Marker	Control	RSL-3	R+4	R+S

Fig.3D **CytC-Cytoplasm**

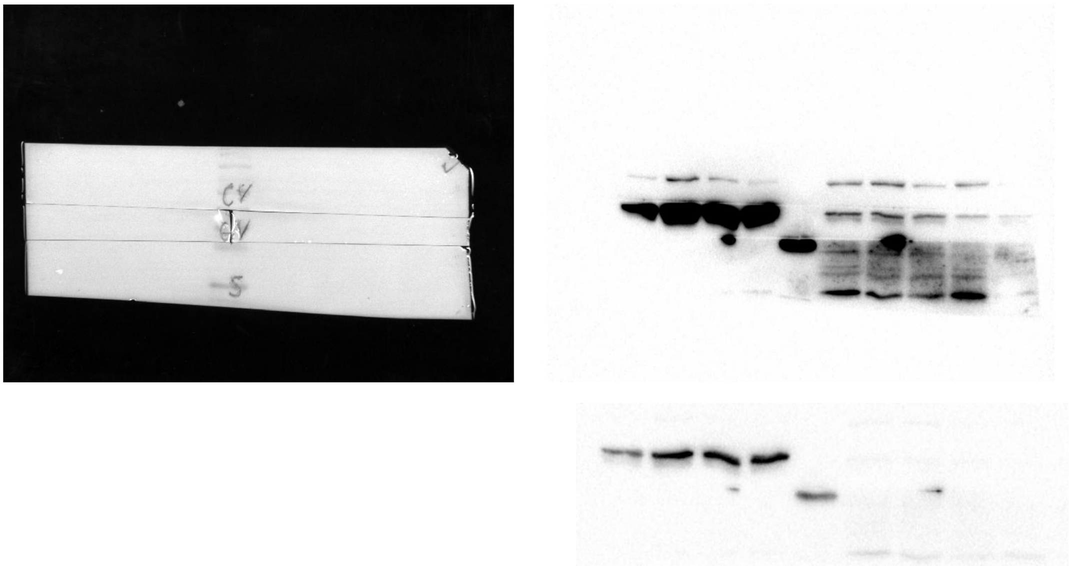

R+S	R+4	RSL-3	Control	Marker	—	—	—	—	—

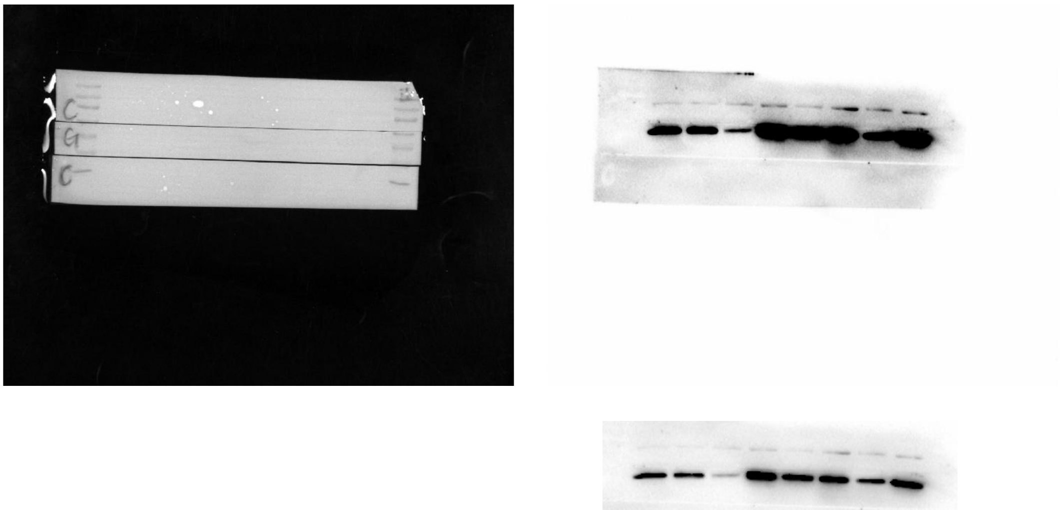

Marker	Control	RSL-3	R+4	R+S	Control	RSL-3	R+4	R+S	Marker
